# RHOA-dependent regulation of mitochondrial remodeling and cell motility in hypoxia-exposed gastric epithelial cells

**DOI:** 10.1242/jcs.263690

**Published:** 2025-07-30

**Authors:** Aranya Pal, Prabin Bawali, Abhisek Brahma, Smruti Ranjan Rana, Rakesh Mohapatra, Debashish Chakraborty, Indrajit Poirah, Supriya Samal, Smaran Banerjee, Duane T. Smoot, Hassan Ashktorab, Asima Bhattacharyya

**Affiliations:** ^1^ School of Biological Sciences, National Institute of Science Education and Research (NISER) Bhubaneswar, An OCC of Homi Bhabha National Institute, P.O. Bhimpur-Padanpur, Via Jatni, Dist. Khurda 752050, Odisha, India; ^2^ Department of Infectious Disease Biology, Institute of Life Sciences, NALCO Square, Chandrasekharpur, Bhubaneswar, Odisha, 751023, India; ^3^ Regional Centre for Biotechnology, 3rd Milestone, Faridabad-Gurgaon Expressway, Faridabad Rd, Faridabad, Haryana, 121001, India; ^4^ Department of Medicine, Meharry Medical Center, Nashville, TN 37208, USA; ^5^ Department of Medicine, Howard University, Washington DC 20060, USA; ^6^ Centre for Interdisciplinary Sciences, National Institute of Science Education and Research, Bhubaneswar, Dist. Khurda 752050, Odisha, India

**Keywords:** Cell motility, Gastric cancer, Lasso-shaped mitochondria, Mitochondrial morphology, RHOA, ROS

## Abstract

Mitochondrial appearance distinctively reflects cellular stress. Hypoxia, one of the most fundamental stressors, drives tumor progression, impacting mitochondrial structure and function. RAS homolog family member A (RHOA), a key regulator of cell motility, is frequently upregulated in response to hypoxia across cancers. However, its behavior under hypoxic conditions in gastric cancer (GC) remains largely unexplored. Additionally, to what extent the role of RHOA in cell motility is mediated through an influence on mitochondrial reshaping is elusive. Here, we show that an elevated RHOA level in GC cells triggers mitochondrial shape changes, from tubular to the stress-associated lasso and donut, correlating with increased reactive oxygen species (ROS). However, *RHOA*-overexpressing cells experiencing hypoxia exhibited increased migration, despite reduced mitochondrial fission and ROS levels. RHO-associated coiled-coil kinase (ROCK) inhibition impaired mitochondrial shape changes, suggesting it has a role in mitochondrial remodeling. These results indicate a unique adaptive response to hypoxia, where RHOA upregulation increases motility and modulates mitochondrial plasticity in GC cells. In summary, RHOA-mediated mitochondrial reshaping might serve as a key regulator in tumor cell adaptation and migration in low-oxygen environments.

## Teaser

**Summary:** Hypoxia-induced modulation of mitochondrial dynamics and cell motility in gastric epithelial cells occurs through RHOA–ROCK signaling.

## INTRODUCTION

Hypoxia is one of the core elements behind the spatial variability in cancer (Ucaryilmaz Metin and Ozcan, 2022). It facilitates proliferation of cancer cells to circumvent growth suppressors and apoptotic signals, thereby dysregulating cellular energetics. Cells undergo a myriad of adaptive transformations in response to hypoxia, culminating in a rapidly proliferating tumor, enhanced chemo-resistance and adverse clinical outcomes (Liu et al., 2016). Central to these adaptations is hypoxia-inducible factor 1 (HIF1), which is one of the main mediators in hypoxic niches (Nayak et al., 2020). The α-subunit of HIF1 (HIF1α) is oxygen labile, whereas the β-subunit (aryl hydrocarbon receptor nuclear translocator, ARNT) remains active regardless of oxygen availability. Together, they form the active transcription factor HIF1 in the hypoxic microenvironment. The human gastric mucosa is subjected to a partial hypoxic milieu, with the severity of hypoxia becoming markedly pronounced in gastric cancer (GC) (Liu et al., 2016). GC ranks among the most prevalent malignant tumors globally, characterized by its pronounced heterogeneity in advanced stages (Mishra et al., 2022). Distinct degrees of differentiation and varying biological behaviors frequently manifest across various subtypes and even within disparate regions of the same neoplasm. Gastric tumors are replete with hypoxic niches (Liu et al., 2020). However, the knowledge gap in GC remains significantly broad, as the volume of research focused on hypoxia is comparatively sparse relative to other extensively studied malignancies (Ucaryilmaz Metin and Ozcan, 2022). Along with the upregulation of HIF1α, many other factors are influenced in hypoxia. RHOA, a principal member of RHO GTPase family is one of the major factors regulated during hypoxia (Orgaz et al., 2014). RHOA functions as a molecular switch regulating many cellular processes, which include cytoskeletal changes, cell motility and signaling events. Hypoxia induces RHOA signaling in hepatocellular carcinoma and promotes vasculogenic mimicry (Zhang et al., 2020b), whereas in lung cancer, hypoxia facilitates RHOA-mediated aggressive cell motility (Kataoka et al., 2019). HIF1α and RHOA upregulation is also found in malignant GC (Wei et al., 2024). In fact, some studies have identified activating mutations in RHOA (e.g. RHOA Y42C mutation) in a subset of diffuse-type GC (Zhang et al., 2020a). In addition, overexpression or hyperactivation of RHOA is associated with increased metastasis and poor prognosis (Zieseniss, 2014). Therefore, these studies highlight that RHOA modulation in hypoxic GC cells might show crucial effects in GC pathogenesis which are yet to be unraveled.

In hypoxia, mitochondria play a central role in regulating cellular responses by affecting ATP production, calcium (Ca^2+^) regulation and apoptosis (Osellame et al., 2012). Mitochondria are highly dynamic organelles, constantly reorganizing themselves in response to altered physiological demands (Chen et al., 2023). Under normal conditions, mitochondria typically adopt a tubular form, but during cellular stress or perturbation, they shift into a variety of striking shapes, each reflecting a unique functional role (Liu and Hajnóczky, 2011). These distinct mitochondrial shapes display diverse responses to substrates, inhibitors and oxidative stress, underscoring their complexity, variability and plasticity (Liu and Hajnóczky, 2011). One of the major outcomes of hypoxia is the generation of reactive oxygen species (ROS) (Tafani et al., 2016). These play a significant role in regulating mitochondrial dynamics, such as fission and fusion (Humphries et al., 2023). Modulations induced by ROS enable the cell to adapt its mitochondrial structure and function in response to varying levels of oxidative stress, linking ROS balance to mitochondrial quality control and cellular homeostasis.

The dynamic regulations are orchestrated by a conserved network of GTPases, which coordinate mitochondrial size and distribution by activating several downstream molecules (Osellame et al., 2012). RHOA, a key GTPase involved in cytoskeletal dynamics, influences downstream kinase molecules and plays a crucial role in cell motility (Matsuoka and Yashiro, 2014). However, the mechanisms by which it regulates mitochondrial dynamics remain less understood. Understanding RHOA-mediated regulation of mitochondrial dynamics in GC might therefore provide a better insight into the regulation of cell movement and invasion.

Our study seeks to offer valuable insights into the mitochondrial adaptations alongside the enhanced cell motility that are driven by RHOA in a hypoxic environment. The findings elucidate how enhanced movement is intricately linked to the dynamics of mitochondrial shape transformation, demonstrating that these alterations are not merely structural but are deeply intertwined with ROS generation and involvement of RHOA and RHO-associated coiled-coil kinase 1 and 2 (hereafter collectively denoted as ROCK). This work contributes to a deeper understanding of how cellular adaptations to hypoxia shape the behavior of cancer cells and shows that RHOA-mediated mitochondrial regulation has potential as a future therapeutic strategy for treatment of advanced GC.

## RESULTS

### Hypoxia increases RHOA level and alters mitochondrial morphology in gastric epithelial cells

**Fig. 1.**
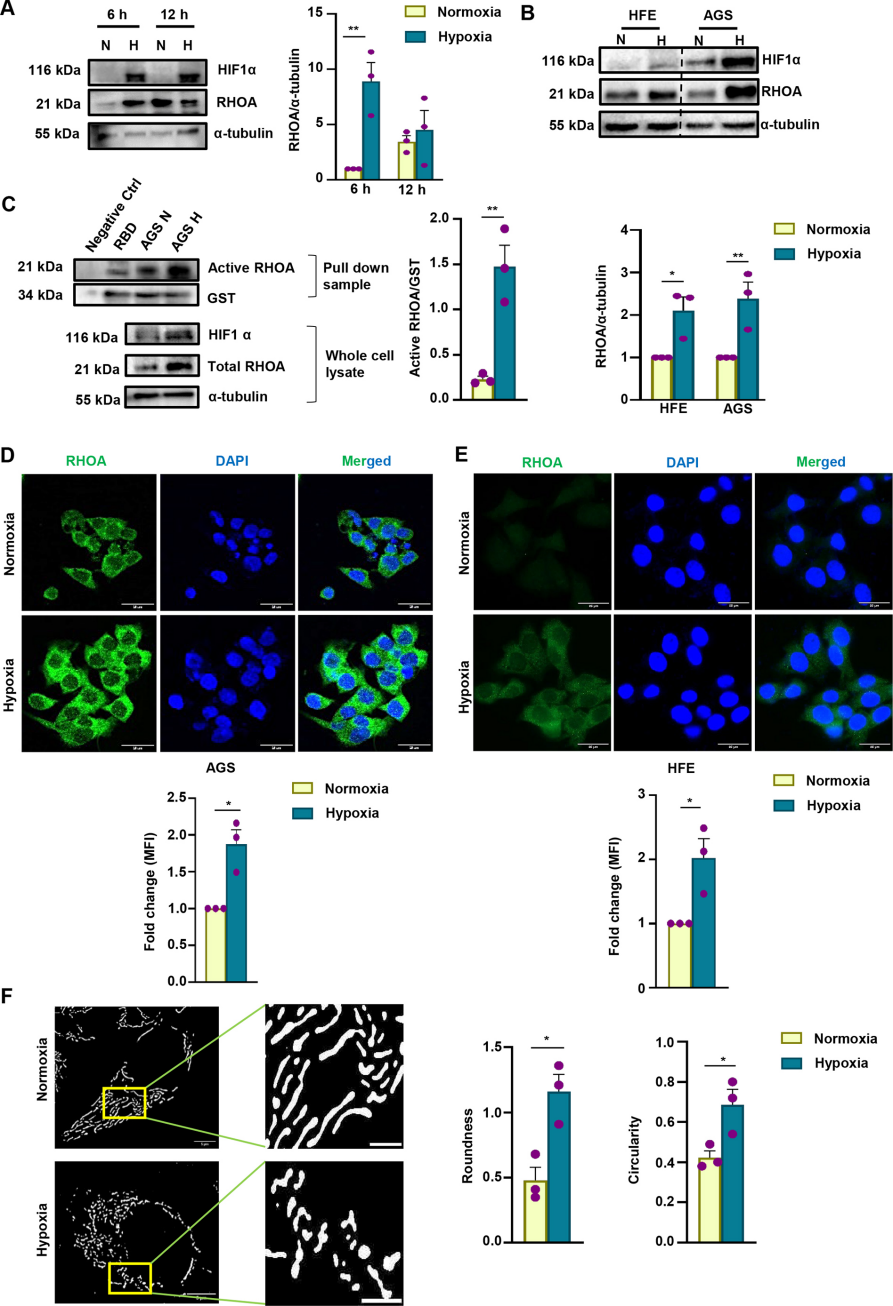
**Hypoxia upregulates RHOA in GECs.** (A) A representative immunoblot showing level of RHOA in whole-cell lysates prepared from normoxic and hypoxic (3% O_2_) (6 h and 12 h) AGS cells. HIF1α was used to denote the status of hypoxia. α-tubulin was used as a loading control. Bar graph displays the optimal time of RHOA upregulation in response of hypoxia (mean±s.e.m., *n*=3), normalized by α-tubulin. Statistical significance was determined by two-way ANOVA followed by Tukey's post hoc analysis. (B) Western blot of whole-cell lysates from HFE145 and AGS depicting the level of RHOA at 6 h of hypoxia exposure. HIF1α was used to denote the status of hypoxia. α-tubulin was used as a loading control. The bar graph below (mean±s.e.m., *n*=3) represents the normalized level of RHOA in response to hypoxia. Statistical significance was determined by two-way ANOVA followed by Tukey's post hoc analysis. (C) Western blot showing active RHOA in pull-down samples prepared from normoxic and hypoxic (3% O_2_) (6 h) AGS cells. Pull-down was carried out using GST-tagged RBD and glutathione affinity beads. GST–RBD was used as loading control to ensure equal RBD binding in each condition. Cell lysates incubated with beads plus RBD (RBD) and without RBD (Negative Ctrl) were used as the negative controls. Input protein (5%) levels in the whole cell lysates are shown in the three lower panels. Bar graphs display activity of RHOA in response of hypoxia (mean±s.e.m., *n*=3), normalized by GST. Statistical significance was determined by unpaired two-tailed Student's *t*-test. (D,E) Micrographs of AGS cells and HFE145 cells exhibiting the fluorescence intensity of RHOA under 6 h of hypoxia. Mean fluorescence intensity (MFI) was quantified and represented as in bar graph (mean±s.e.m., *n*=3). Statistical significance was determined by unpaired two-tailed Student's *t*-test. (F) Super-resolution microscopy was performed, and images of mitochondria from hypoxia or normoxia-exposed AGS cells were acquired after thresholding and masking with the help of Mitochondria Analyzer. The bar graphs (mean±s.e.m., *n*=3) represent the quantitative measurement of roundness and circularity. 30 cells were analyzed from three independent experiments. Statistical significance was determined by unpaired two-tailed Student's *t*-tests. In all graphs, individual data points were represented by purple dots. *P<0.05, **P<0.01. N, normoxia; H, hypoxia. Scale bars: 25 μm (D,E), 5 μm (F, main image), 2 μm (F, inset).

Hypoxia and RHOA signaling provide essential axes in GC progression (Nayak et al., 2020; Schaefer et al., 2023). The current study sought to evaluate the influence of hypoxia on RHOA in gastric epithelial cells (GECs). Here, we need to remember that the median oxygenation in untreated tumors falls between ∼0.3% and ∼4.2% oxygen (O_2_), with most tumors exhibiting a level of ≤3% (de Vos et al., 2022; Zheng et al., 2015). Also, each tissue experiences varying levels of oxygenation based on differing conditions. Human gastric tissue is exposed to partial O_2_ pressures of 60–77 mm Hg (7999.34–10,265.82 Pa; corresponding to 8–10% O_2_) from the luminal side to the muscular wall. A study from our group (Poirah et al., 2025) has established that 3% O_2_ is hypoxic for GECs. To identify the temporal profile of RHOA protein upregulation under hypoxia, gastric adenocarcinoma cells, such as AGS, were exposed to 3% O_2_ for 6 h and 12 h. Representative western blot results (*n*=3) confirmed that RHOA level significantly increased at 6 h of hypoxia when compared with those in normoxic cells (Fig. 1A). The establishment of hypoxia was verified by HIF1α protein level. Accordingly, all the subsequent experiments were conducted at 3% O_2_ for 6 h, unless described differently. A pan-cancer analysis based on The Cancer Genome Atlas (TCGA) in Tumor Immune Estimation Resource 2.0 (TIMER2.0; http://timer.cistrome.org/; Li et al., 2020) has also shown that there is significant upregulation of *RHOA* mRNA expression across 12 of the 34 tumor types compared to their equivalent normal counterparts, with stomach adenocarcinoma (STAD) being one of them (Fig. S1A). In addition, the University of ALabama at Birmingham CANcer data analysis portal (UALCAN; https://ualcan.path.uab.edu/; Chandrashekar et al., 2017) showed an elevated expression of *RHOA* in STAD tumors (Fig. S1B). Increased *RHOA* expression was found to be associated with advanced tumor stages (Fig. S1C). Correlation analysis using the Gene Expression Profiling Interactive Analysis web server (GEPIA2; http://gepia2.cancer-pku.cn/; Tang et al., 2019) revealed a significant association between *RHOA* and *HIF1A*, with a correlation coefficient of 0.69 (Fig. S1D). The survival analysis, calculated using Kaplan–Meier plotter (https://kmplot.com/analysis/; Lánczky and Győrffy, 2021), also indicated poor probable survival rate upon increased RHOA protein (Fig. S1E). Apart from AGS cells, RHOA was found to be expressed in 41 other GC cell lines as acquired from The Human Protein Atlas (HPA; https://www.proteinatlas.org/; Thul and Lindskog, 2018) (Fig. S1F). Next, we were intrigued to unravel whether noncancerous GECs (HFE145) behave in the same manner as AGS cells. Interestingly, not only AGS but also HFE145 cells showed a significant RHOA increase after 6 h hypoxia exposure (Fig. 1B) indicating that this event is common in normal and cancerous GECs. Given that RHOA is a small GTPase, it has a GTP-bound (active) and a GDP-bound (inactive) state. Besides measuring the total RHOA protein level, we performed an activity assay to estimate the amount of active RHOA in AGS cells at 6 h of hypoxia exposure. RHOA was significantly more active in hypoxic cells as compared to that of normoxic cells (Fig. 1C). Next, confocal microscopy (*n*=3) was performed, showing that the RHOA level was significantly higher in hypoxic AGS cells than in normoxic cells, as indicated by the mean fluorescence intensity (MFI) (Fig. 1D), which corroborated our earlier observations (Fig. 1A,B). Immunofluorescence microscopy of HFE145 also matched with the AGS response (Fig. 1E).

Cellular oxygenation status can regulate the mitochondrial dynamics. Previous studies have shown that under normoxia, mitochondria assemble into elongated networks, whereas in hypoxia, mitochondria appear as fragmented organelles (Fuhrmann and Brüne, 2017). In order to investigate the effect of hypoxia exposure on mitochondrial morphology, AGS cells were transfected with pDsred2-Mito construct (encoding a mitochondria-targeting sequence from the subunit VIII of human cytochrome *c* oxidase) followed by hypoxia or normoxia exposure. Super-resolution microscopy (of 30 cells, *n*=3) showed that the tubuloreticular morphology of mitochondria was altered significantly and reduced in size after hypoxic exposure, as indicated by the measurements of roundness and circularity (Fig. 1F). Collectively, the results in Fig. 1 signify that hypoxia has prominent roles in upregulating RHOA activity and modulating mitochondrial morphology in GECs.

### Mitochondrial dynamics in hypoxic GECs is modulated by RHOA

**Fig. 2.**
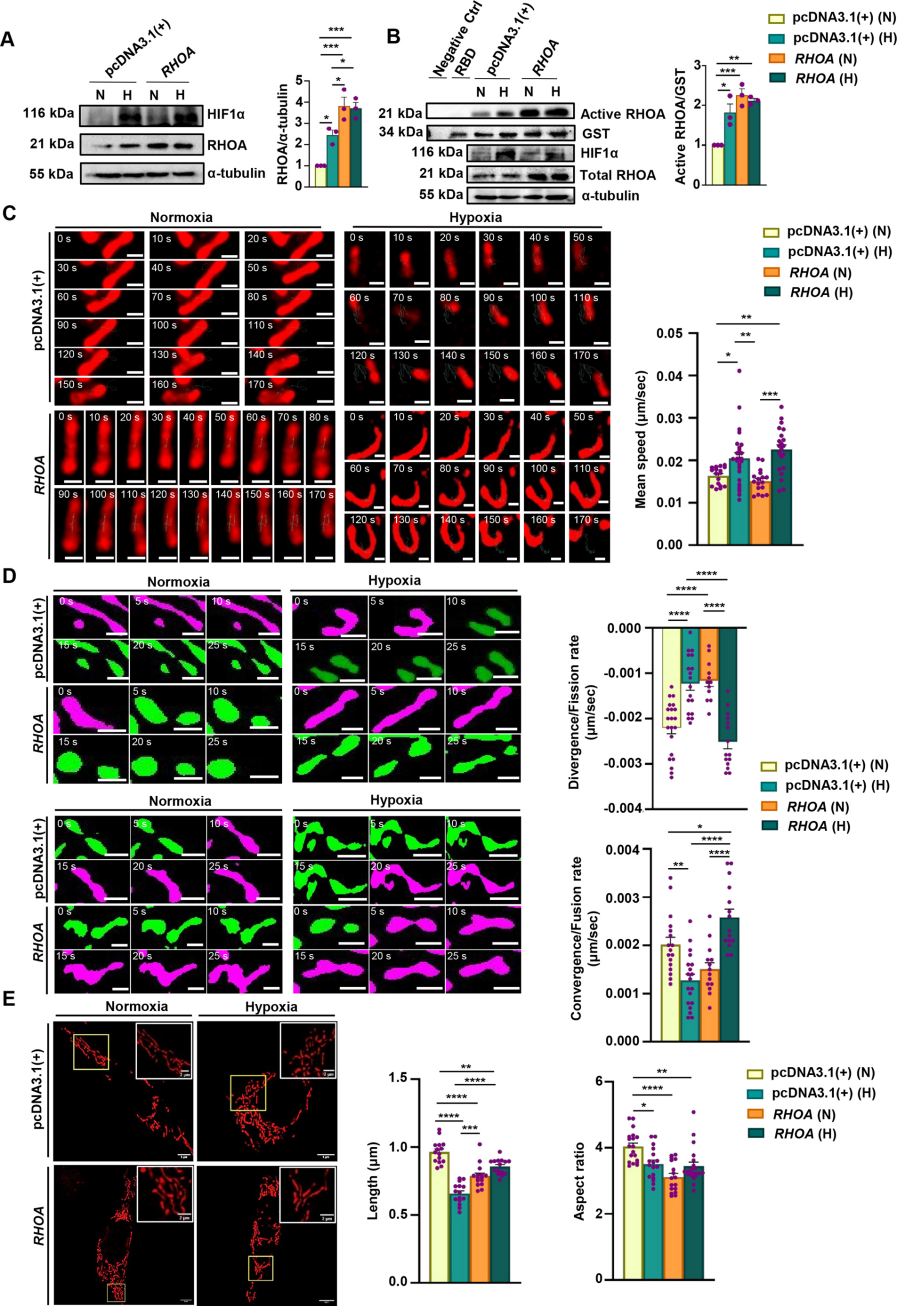
**RHOA and hypoxia induce changes in mitochondrial dynamics.** (A) Western blot showing RHOA level in RHOA- and empty vector-stable cells subjected to hypoxia or normoxia. HIF1α was probed to denote the status of hypoxia. α-tubulin was used as a loading control. The quantification of protein relative to α-tubulin is shown in the graph (mean±s.e.m., *n*=3). Statistical significance was determined by two-way ANOVA followed by Tukey's post hoc analysis. (B) Representative blot showing level of active RHOA in pull-down samples prepared from RHOA- and empty vector-stable cells subjected to normoxic and hypoxic conditions. Pull-down was carried out using GST-tagged RBD and glutathione affinity beads. GST-RBD was used as loading control to ensure equal RBD in each condition. Cell lysates incubated with beads plus RBD (RBD) and without RBD (Negative Ctrl) were used as the negative controls. The quantification of active protein relative to GST has been shown in the graph (mean±s.e.m., *n*=3). Input lysates (5%) are shown in the lower panels. Statistical significance was determined by two-way ANOVA followed by Tukey's post hoc analysis. (C) Time-lapse images showing mitochondrial mean speed in RHOA- and empty vector-stable AGS cells under hypoxia and normoxia. A track mark of an individual mitochondrion has been shown in blue color (Movie 1). The bar graph (mean±s.e.m., *n*=3) represents changes in mean speed (µm/s) of mitochondria due to *RHOA* and empty vector transfection. *n*>100 mitochondria/group from three independent repeats. Statistical significance was determined by one-way ANOVA followed by Tukey's post hoc analysis. (D) Time-lapse micrographs depicting the fission (upper panel) and fusion (lower panel) rate (µm/s) of a single mitochondrion exposed to hypoxia and normoxia in RHOA- and empty vector-stable cells. Mitochondria were tracked for 25 s for each group. Fused mitochondria are indicated with magenta (pseudocolor) and after fission they are represented in green (pseudocolor). *n*>100 mitochondria/group from three independent repeats were analyzed. Bar graphs (mean±s.e.m., *n*=3) represents the divergence (fission) rate, convergence (fusion) rate (µm/s) of mitochondria due to *RHOA* and empty vector transfection. Statistical significance was determined by one-way ANOVA followed by Tukey's post hoc analysis. (E) Representative micrographs depicting mitochondria of a single AGS cell transfected with *RHOA* and empty vector subjected to hypoxic and normoxic exposure. The structural changes of mitochondria is shown in the insets. Objective magnification: 63×. Graphical representations (mean±s.e.m., *n*=3) depict the change in length (µm) and aspect ratio in RHOA- and empty vector-stable cells exposed to hypoxia and normoxia. *n*>100 mitochondria/group from three independent repeats. Statistical significance was determined by one-way ANOVA followed by Tukey's post hoc analysis. In all graphs, individual data points were represented by purple dots. *P<0.05, **P<0.01, ***P<0.001, ****P<0.0001. N, normoxia; H,hypoxia; empty vector, pcDNA3.1(+). Scale bars: 0.5 μm (C,D), 5 µm (E, main images), 2 µm (E, insets).

Mitochondrial dynamics and morphology in GC remain largely uncharted territories. To explore the effect of RHOA in the mitochondrial dynamics, *RHOA*-overexpressing stable AGS cells (hereafter denoted as RHOA-stable cells) were established. When compared with the empty vector pcDNA3.1(+)-transfected AGS cells (hereafter denoted as empty vector-stable cells), the RHOA-stable cells showed significantly increased RHOA protein (Fig. S2A). Further studies were undertaken to examine whether hypoxia exposure could alter RHOA level in RHOA-stable cells. Data showed a significant increase in RHOA in empty vector-stable cells after hypoxia exposure compared to normoxia-exposed cells. *RHOA* overexpression led to an increase in RHOA protein level; however, the hypoxic condition did not further elevate RHOA level in RHOA-stable cells beyond the level observed in their normoxic counterparts (Fig. 2A). Not only the total protein level but also the activity of RHOA significantly increased in hypoxic empty vector-stable cells and normoxic and hypoxic RHOA-stable cells (Fig. 2B).

To explore the dynamics of mitochondria in RHOA-stable cells under normoxia and hypoxia, RHOA and empty vector-stable cells were transfected with pDsRed2-Mito. Mitochondrial dynamics were analyzed using live cell super-resolution microscopy over a 3 min window, capturing frames at 5 s intervals. The key features of mitochondria were explored using Nellie (Lefebvre et al., 2025). To quantify the mean speed, more than 100 mitochondria were tracked in time-lapse images, and tracks of an individual mitochondrion from the hypoxia or normoxia-exposed empty vector- or RHOA-stable cells were represented with blue track marks (associated with Movie 1) to illustrate the trajectory change of the mitochondrion in each 10 s time frame (Fig. 2C). Data revealed that mitochondrial motility varied significantly, from 0.0123–0.0189 μm/s in normoxic empty vector-stable cells, 0.0107–0.0211 μm/s in hypoxic empty vector-stable cells, 0.0115–0.0203 μm/s in normoxic RHOA-stable cells and 0.012–0.04 μm/s in hypoxia-exposed RHOA-stable cells. Nonetheless, among all treatment groups, RHOA-stable cells under hypoxia showed the fastest speed (Fig. 2C). The mitochondrial speed for the four cell groups can be found in Movie 1. In addition, the continuous fluctuations in speed in the 3 min time frame also corroborated the results (Fig. S2B). Next, mitochondrial fission, or the rate of divergence, was analyzed to assess the effect of RHOA on altered fission rates. In Fig. 2D, the fission process of a single mitochondrion over a 0–25 s timeframe has been illustrated for the four cell groups, where the fused mitochondrion is shown in magenta and the segregated one, post-fission, in green. In normoxic condition, RHOA-stable cells initiated fission rapidly at 5 s, whereas empty vector-stable cells exhibited a delayed fission at 15 s. Interestingly, under hypoxic conditions, RHOA-stable cells exhibited fission at 15 s, compared to 10 s in empty vector-stable cells. Overall, RHOA-stable cells in normoxic conditions showed a significantly higher fission rate than other transfection groups with the exception of hypoxic empty vector-stable cells (Fig. 2D). The fission of the mitochondria is shown in Movie 2. Similarly, the rate of convergence or fusion process was represented by an individual mitochondrion tracked for 0–25 s for both RHOA- and empty vector-stable cells. Under normoxia, RHOA-stable cells showed fusion at 15 s, whereas empty vector-stable cells showed an early fusion at 10 s. Under hypoxia, RHOA-stable cells exhibited rapid convergence at 5 s, whereas empty vector-stable cells showed the most delayed fusion, at ∼20 s. Collectively, RHOA-stable cells under hypoxic condition showed a significantly higher fusion rate than other groups (Fig. 2D). The fusion of mitochondria for the four groups is illustrated in Movie 3. In addition to the fission–fusion dynamics, mitochondrial length was also evaluated. Empty vector cells in normoxia were the longest, followed by RHOA-stable cells under both hypoxic and normoxic conditions (Fig. 2E). Empty vector-stable cells in hypoxia displayed the shortest mitochondrial length. The aspect ratio was significantly higher in normoxic conditions, suggesting that hypoxia tends to produce punctated mitochondria, whereas normoxia favors the formation of an elongated and tubuloreticular network. Continuous changes in the length, convergence and divergence in hypoxia and normoxia-exposed RHOA and empty vector-stable cells for the 3 min time course are provided in the plots shown in Fig. S2C–E, respectively.

### Impact of RHOA and hypoxia on mitochondrial morphology

Mitochondrial morphologies are associated with a plethora of physiological and pathological conditions, including stress, but only in a few instances have there been studies to functionally link the changes in morphology with a phenotype or cell behavior (Ahmad et al., 2013; Sun et al., 2018; Zhao et al., 2013).

**Fig. 3.**
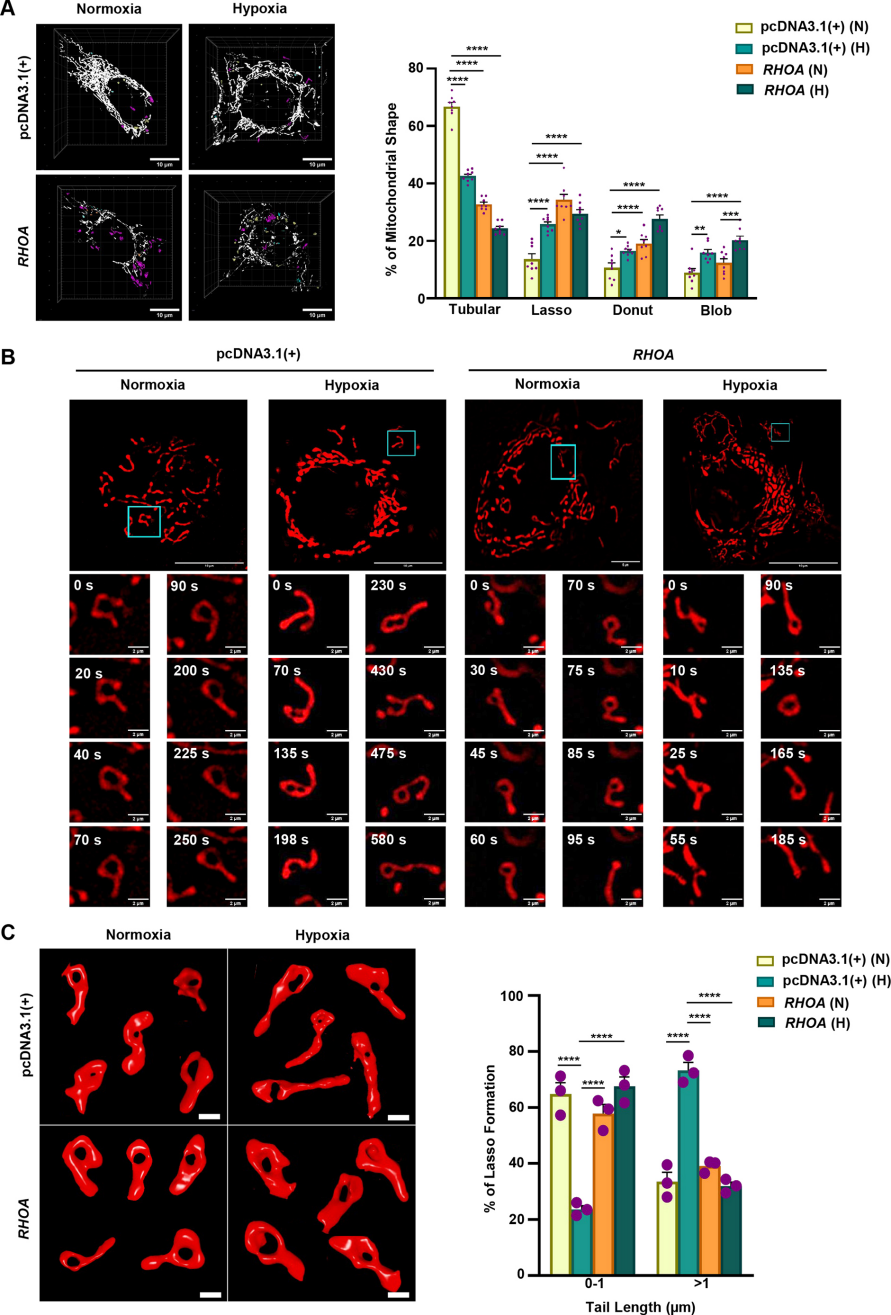
**Increased RHOA level in GECs promotes lasso formation.** (A) 3D images showing the occurrence of tubular (white), lasso (magenta), donut (blue) and blob (yellow) shaped mitochondria in RHOA- and empty vector-stable AGS cells with or without hypoxia exposure. Objective magnification: 63×. The bar graph (mean±s.e.m., *n*=3) depicts the percentage of above-mentioned shapes in the four transfected groups. *n*>100 mitochondria/group. Statistical significance was determined by two-way ANOVA followed by Tukey's post hoc analysis. (B) Time-lapse super-resolution micrographs represented the formation of lasso-shaped mitochondria in RHOA- and empty vector-stable cells exposed to hypoxia and normoxia. *n*>100 mitochondria/ group from three independent repeats. (C) Representative image showing 3D models of varied lassoes formed in RHOA- and empty vector-stable cells exposed to hypoxia and normoxia. Bar graph (mean±s.e.m., *n*=3) shows the percentage of all mitochondria observed that had lasso formation with tail lengths ranging from 0–1 µm and >1 µm in the above-mentioned transfected groups. *n*>100 mitochondria/group. Statistical significance was determined by two-way ANOVA followed by Tukey's post hoc analysis. In all graphs, individual data points are represented by purple dots. *P<0.05, **P<0.01, ***P<0.001, ****P<0.0001. N, normoxia; H, hypoxia; empty vector, pcDNA3.1(+). Scale bars: 10 μm (A), 5 µm (B, main images), 2 µm (B, magnifications), 0.5 μm (C).

In this study, mitochondrial morphology was explored in response to hypoxia and *RHOA* overexpression. To achieve this, pDsRed2-Mito-transfected RHOA-stable and empty vector-stable cells were exposed to hypoxia and normoxia. Super-resolution microscopy revealed four distinct mitochondrial morphologies, namely tubular, lasso (loop with tail), donut and blob (Fig. 3A). Empty vector-stable cells predominantly exhibited tubular mitochondria under both normoxic and hypoxic conditions, with a significantly higher occurrence in normoxia. In contrast, RHOA-stable cells under normoxia showcased a striking abundance of lasso-shaped mitochondria. The donut-shaped mitochondria were significantly more abundant in RHOA-stable cells under hypoxia, whereas the blob shape emerged mostly under hypoxic conditions. Microscopy study exhibited that RHOA level was significantly higher in association with the lasso shape (Fig. S3A), which corroborated our above observations. Although the donut form has been extensively studied as a mitochondrial stress marker, the intermediate lasso structure remains an intriguing, yet underexplored, phenomenon in cancer research. The lasso structure is mostly observed during oxidative stress (Liu and Hajnóczky, 2011), exposure to cold temperature (Quiring et al., 2022) and in response to carbonyl cyanide p-(trifluoromethoxy)phenylhydrazone (FCCP) (Liu and Hajnóczky, 2011) or carbonyl cyanide m-chlorophenyl hydrazone (CCCP) treatment (Preminger and Schuldiner, 2024), where it typically forms as a result of tip-to-middle fusion. To further understand this enigmatic shape in our context, more than 100 mitochondria were tracked for 10 min (at 5 s intervals) in both RHOA- and empty vector-stable cells in hypoxic and normoxic conditions. Live imaging revealed a dynamic and captivating process of this shape transformation. Over a 10 min duration, the frequency of lasso formation notably increased in normoxic RHOA-stable cells and hypoxic empty vector-stable cells (Fig. S3B). There was considerable variation in the formation of the lasso shape, as illustrated in Fig. 3B. In empty vector-stable cells under normoxia, lasso formation occurred in a tip-to-middle fashion, whereas in empty vector-stable cells under hypoxia and RHOA-stable cells under normoxia, the transformation began from a Y-shaped structure, which gradually fused at the tips to form the classic lasso within a min. In hypoxia-exposed RHOA-stable cells the lasso shape was less stable, constantly breaking and reforming in response to the hypoxic stress level of cell, occasionally transitioning into the typical donut shape. Furthermore, the formation and stability of the lasso were not uniform between empty vector- and RHOA-stable cells. Despite being common in the aforementioned situations, these formations of lasso are not condition specific. Formation of various lasso types is shown in Movie 4. 3D visualizations of this intriguing structure are shown in Fig. 3C to provide a clear understanding of its structural parameters. Quantitative analysis revealed that both normoxic and hypoxic RHOA-stable cells, as well as normoxic empty vector-stable cells, exhibited shorter tail lengths (0–1 μm) in their lasso-shaped mitochondria compared to those in hypoxic control cells. In contrast, the empty vector-stable cells under hypoxia displayed significantly longer tail lengths, exceeding 1 μm, when compared to other transfection groups. Apart from the 3D models displayed in Fig. 3C, other types of lassos also appeared (Fig. S3C). In addition, although less frequent in our conditions, donut shapes also appeared (see 3D models in Fig. S3D). The striking presence of the lasso shape in RHOA-stable cells and hypoxic empty vector-stable cells, both of which are characterized by heightened RHOA level (Fig. S3A), clearly demonstrates that RHOA is pivotal in orchestrating mitochondrial shape transformations in GECs.

### Suppression of *HIF1A* and *RHOA* significantly impacts mitochondrial dynamics

**Fig. 4.**
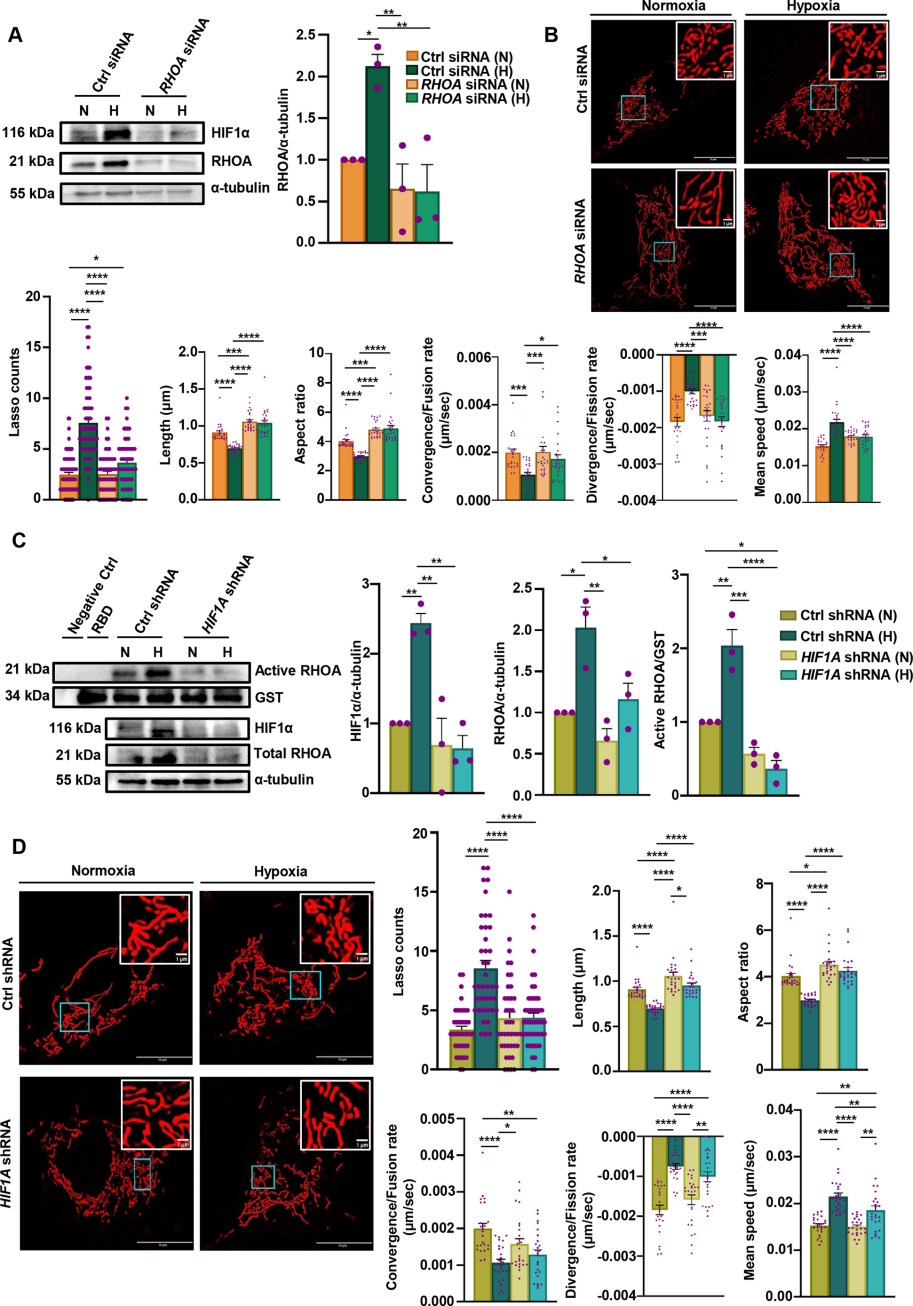
**Suppression of *RHOA* and *HIF1α* alters mitochondrial dynamics and morphology in hypoxic GECs.** (A) Western blot showing RHOA level in AGS cells transfected with control siRNA and *RHOA* siRNA subjected to hypoxia or normoxia. HIF1α was probed to denote the status of hypoxia. α-tubulin was used as a loading control. The quantification of protein relative to α-tubulin is shown in the graph to the right (mean±s.e.m., *n*=3). Statistical significance was determined by two-way ANOVA followed by Tukey's post hoc analysis. (B) Representative micrographs depicting mitochondria of a single AGS cell transfected with *RHOA* siRNA and control siRNA subjected to hypoxia and normoxia. The structural changes of mitochondria are highlighted in the insets. Objective magnification: 63×. The six graphs (mean±s.e.m., *n*=3) shown below depict the numbers of lasso-shaped mitochondria formed (per cell), changes in length (µm), aspect ratio, convergence (fusion) rate (µm/s), divergence (fission) rate (µm/s) and mean speed (µm/s) in *RHOA* siRNA- and control siRNA-transfected AGS cells exposed to hypoxia and normoxia. *n*>100 mitochondria/group. Statistical significance was determined by two-way ANOVA followed by Tukey's post hoc analysis. For lasso shape analysis, one-way ANOVA followed by Tukey's post hoc analysis was used. (C) Representative blot showing active RHOA in pull-down samples prepared from normoxic and hypoxic *HIF1A* shRNA- and control shRNA-stable AGS cell lysates. GST-RBD was used to ensure equal RBD in each condition. Cell lysates incubated with beads plus RBD (RBD) and without RBD (Negative Ctrl) were used as the negative controls. The quantification of active protein relative to GST is shown in the graphs (mean±s.e.m., *n*=3). In addition, total RHOA and HIF1α was assessed in the input. α-tubulin was used as a loading control. The quantification of total HIF1α and RHOA proteins relative to α-tubulin was performed and is shown in the graphs (mean±s.e.m., *n*=3). Statistical significance was determined by two-way ANOVA followed by Tukey's post hoc analysis. (D) Micrographs showing mitochondria of *HIF1A* shRNA- and control shRNA-stable cells subjected to hypoxic and normoxic exposure. The structural changes of mitochondria are shown in the insets. Objective magnification: 63×. Representative graphs (mean±s.e.m., *n*=3) indicate numbers of lasso-shaped mitochondria formed (per cell), changes in length (µm), aspect ratio, convergence (fusion) rate (µm/s), divergence (fission) rate (µm/s) and mean speed (µm/s) upon *HIF1A* suppression in hypoxia and normoxia. *n*>100 mitochondria/group. Statistical significance was determined by two-way ANOVA followed by Tukey's post hoc analysis and for quantification of mitochondrial shapes one-way ANOVA has been used. In all graphs, individual data points are represented by purple dots. *P<0.05, **P<0.01, ***P<0.001, ****P<0.0001. N, normoxia; H, hypoxia; Ctrl, Control. Scale bars: 10 µm (B,D, main images), 1 µm (B,D, insets).

Given that mitochondrial dynamics and morphological changes were drastic in RHOA-stable cells under normoxia and hypoxia, *RHOA* siRNA was used to explore whether these mitochondrial changes would be restored after *RHOA* suppression. RHOA protein level was significantly reduced following transient transfection with *RHOA*-specific siRNA compared to what was seen in control siRNA-transfected AGS cells (Fig. 4A). In addition, HIF1α was also downregulated even under hypoxia in RHOA-suppressed cells, indicating a RHOA–HIF1α interplay under hypoxia. Mitochondrial morphology, particularly the lasso shape, was analyzed upon *RHOA* suppression. As compared to the hypoxic control siRNA cells, the number of lasso-shaped mitochondria per cell was significantly decreased in *RHOA* siRNA cells under normoxia and hypoxia (Fig. 4B). Apart from morphological changes, alteration of mitochondrial dynamics was also observed. Under normoxia and hypoxia, *RHOA* siRNA cells showed a significant increase in length, aspect ratio and convergence of mitochondria as compared to that in hypoxic control siRNA cells. However, divergence and mean speed were significantly reduced in normoxic and hypoxic *RHOA* siRNA cells as compared to the hypoxic control siRNA cells (Fig. 4B). Above all, *RHOA* suppression altered the complete mitochondrial dynamics, with these cells exhibiting a much more fused mitochondrial morphology, confirming the role of RHOA behind these mitochondrial regulations.

As already established, RHOA and hypoxia both were crucial in mitochondrial alterations. However, it was not clear whether HIF1α was solely responsible for these mitochondrial alterations. In order to verify the effect of HIF1α on RHOA activity and mitochondrial dynamics, *HIF1A* short hairpin (sh)RNA-expressing (hereafter denoted *HIF1A* siRNA-stable cells) and control shRNA-stable cells were used (Bhattacharyya et al., 2010; Rath et al., 2015). HIF1α was downregulated in *HIF1A* shRNA-stable cells in normoxia as well as hypoxia (Fig. 4C). RHOA level was significantly downregulated in *HIF1A* shRNA-stable cells under both normoxia and hypoxia, relative to that in hypoxic control shRNA-stable cells (Fig. 4C). Not only the total protein level but the level of active RHOA was also significantly reduced in *HIF1A* shRNA-stable cells as compared to its control shRNA groups under both normoxia and hypoxia. The effect of decreased RHOA activity on mitochondrial morphology and dynamics was next studied. As compared to control shRNA-stable cells, the number of lasso-shaped mitochondria per cell was significantly decreased in *HIF1Α* shRNA-stable cells under both hypoxia and normoxia (Fig. 4D). Additionally, under both normoxic and hypoxic conditions, *HIF1A* shRNA-stable cells exhibited significantly increased mitochondrial length and aspect ratio, along with reduced mean speed, compared to hypoxia-exposed control shRNA-stable cells. However, under normoxia, the increase in convergence and decrease in divergence noticed in *HIF1A* shRNA-stable cells were statistically significant when compared to control shRNA-stable cells in hypoxia, but there were no significant differences between hypoxic *HIF1A* shRNA- and hypoxic control shRNA-stable cells. Therefore, HIF1α is one of the major factors responsible for RHOA activation and hypoxia-driven mitochondrial changes.

### RHOA differentially modulates ROS and mitochondrial dynamics in normoxic and hypoxic GECs

**Fig. 5.**
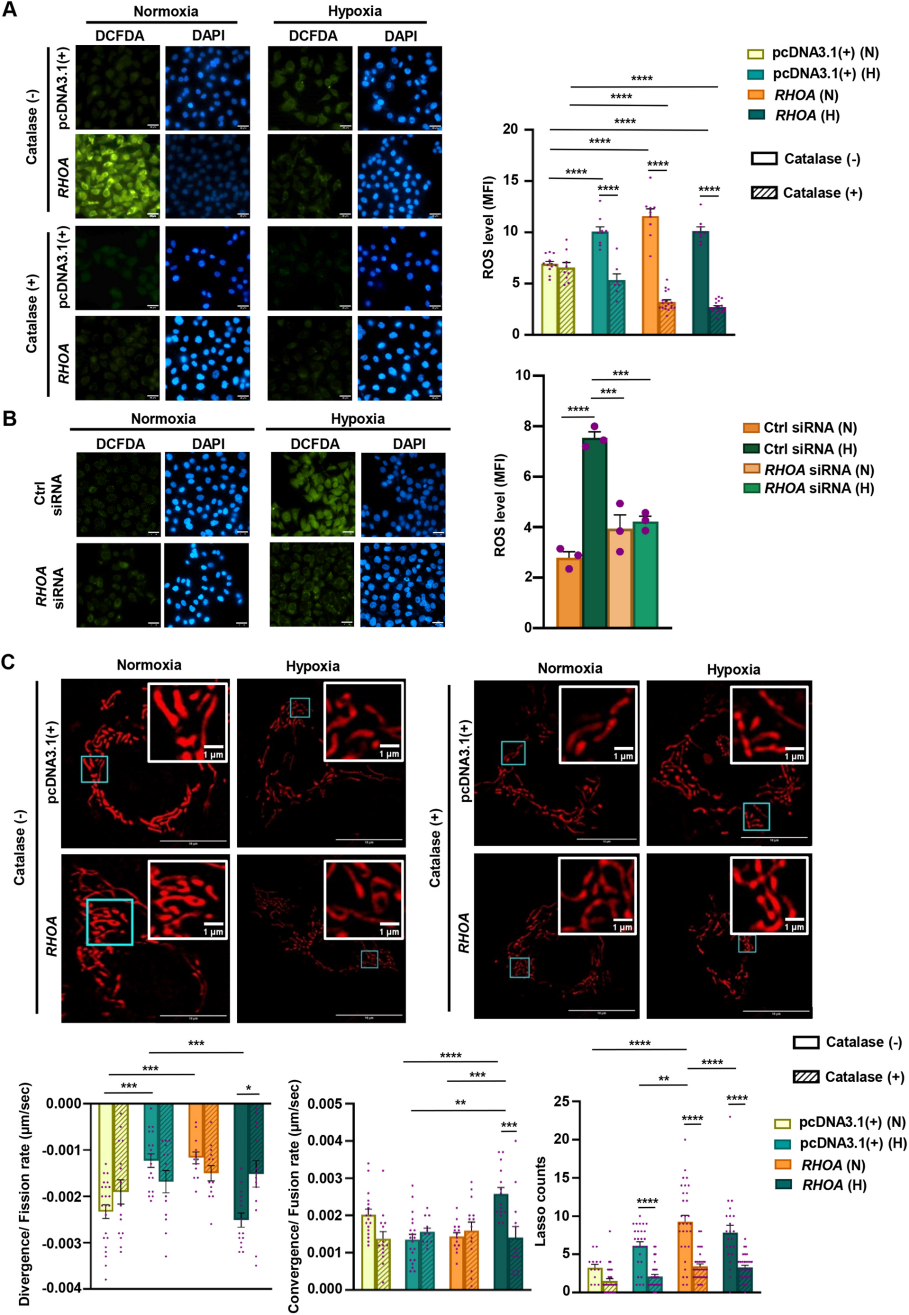
**RHOA differently regulates ROS and mitochondrial dynamics in hypoxic GECs.** (A) Fluorescence microscopy results comparing ROS generation in empty vector- and RHOA-stable cells under normoxic and hypoxic exposure in the presence or the absence of catalase. DCFDA was used for ROS detection and DAPI was used to stain nuclei. Objective magnification: 63×. The bar graph (mean±s.e.m., *n*=3) shows ROS level as assessed by MFI. Statistical significance was determined by two-way ANOVA followed by Tukey's post hoc analysis. (B) Microscopy images showing ROS levels in *RHOA* siRNA- and control siRNA-transfected cells. DCFDA was used for ROS detection and DAPI was used to stain nuclei. Objective magnification: 63×. The bar graph (mean±s.e.m., *n*=3) shows ROS level as assessed by MFI. Statistical significance was determined by two-way ANOVA followed by Tukey's post hoc analysis. (C) Super-resolution microscopic images illustrating mitochondrial dynamics in RHOA- and empty vector-stable cells due to catalase treatment. The structural changes of mitochondria are shown in the insets. Objective magnification: 63×. Representative graphs (mean±s.e.m., *n*=3) showing divergence, convergence and the numbers of lasso-shaped mitochondria formed (per cell). *n*>100 mitochondria/cell group. Statistical significance was determined by two-way ANOVA followed by Tukey's post hoc analysis. In all graphs, individual data points were represented by purple dots. *P<0.05, **P<0.01, ***P<0.001, ****P<0.0001. N, normoxia; H, hypoxia; empty vector, pcDNA3.1(+); MFI, mean fluorescent intensity; Ctrl, Control. Scale bars: 25 μm (A,B), 10 µm (C, main images), 1 µm (C, insets).

Previous studies have revealed that ROS can regulate mitochondrial shape changes, primarily promoting mitochondrial fission and fragmentation (Ahmad et al., 2013). This regulation is important for cancer cells, as mitochondrial fragmentation is associated with enhanced energy production, cellular survival and adaptability in the tumor microenvironment. To explore ROS production, RHOA-stable cells and empty vector-stable cells were subjected to hypoxia and normoxia, and were treated with 1 μM dichlorodihydrofluorescein diacetate (DCFDA) solution for 1 h. Immunofluorescence microscopy revealed that empty vector-stable cells under hypoxia exhibited significantly higher ROS levels as compared to normoxic cells. In normoxic RHOA-stable cells, a further increased ROS level was noticed but a downregulation occurred with hypoxia exposure (Fig. 5A). As in hypoxic cells, hydrogen peroxide (H_2_O_2_) is one of the prime ROS (Schieber and Chandel, 2014), cells were exposed to 350 units of catalase/ml for 1 h prior to their exposure to hypoxia or normoxia as per our in-house established protocol (Dixit et al., 2022). Significant downregulation of ROS was noticed in all catalase-treated groups except for normoxic empty vector-stable cells. To validate the role of RHOA in ROS regulation, the status of ROS generation was studied in *RHOA* siRNA-transfected cells. *RHOA*-suppressed cells showed a significant decrease in ROS generation as compared to the hypoxic control siRNA cells under normoxia and hypoxia (Fig. 5B). To quantify the mitochondrial dynamics and morphological changes in response to this ROS modulation, RHOA- and empty vector-stable cells were transfected with pDsRed2-Mito and subjected to hypoxia and normoxia. The cells were treated with or without catalase and mitochondrial dynamics was tracked for 3 min by super-resolution microscopy. Mitochondria of the RHOA-stable and empty vector-stable cells are displayed in Fig. 5C. Normoxic empty vector-stable cells exhibited a lower mitochondrial convergence or fusion rate upon ROS reduction, whereas hypoxic empty vector-stable cells demonstrated increased fusion following catalase intervention, as compared to their respective untreated controls. RHOA-stable cells in normoxia also showed a recovery in fusion rates with ROS downregulation. These observations are consistent with an increase in mitochondrial divergence or fission rates among normoxic empty vector-stable cells and decreased fission rates in hypoxic empty vector- and normoxic RHOA-stable cells after catalase treatment. Although these changes in fusion and fission rates were not statistically significant among the three treated and untreated groups (normoxic and hypoxic empty vector-stable cells and normoxic RHOA-stable cells), however, significantly decreased fusion and increased fission were observed in hypoxic RHOA-stable cells following catalase treatment. The number of lasso occurrences also significantly decreased in catalase-treated cells compared to untreated cells except for the normoxic empty vector.

### Role of RHOA and hypoxia in regulating ROCK-dependent mitochondrial integrity and GEC motility

**Fig. 6.**
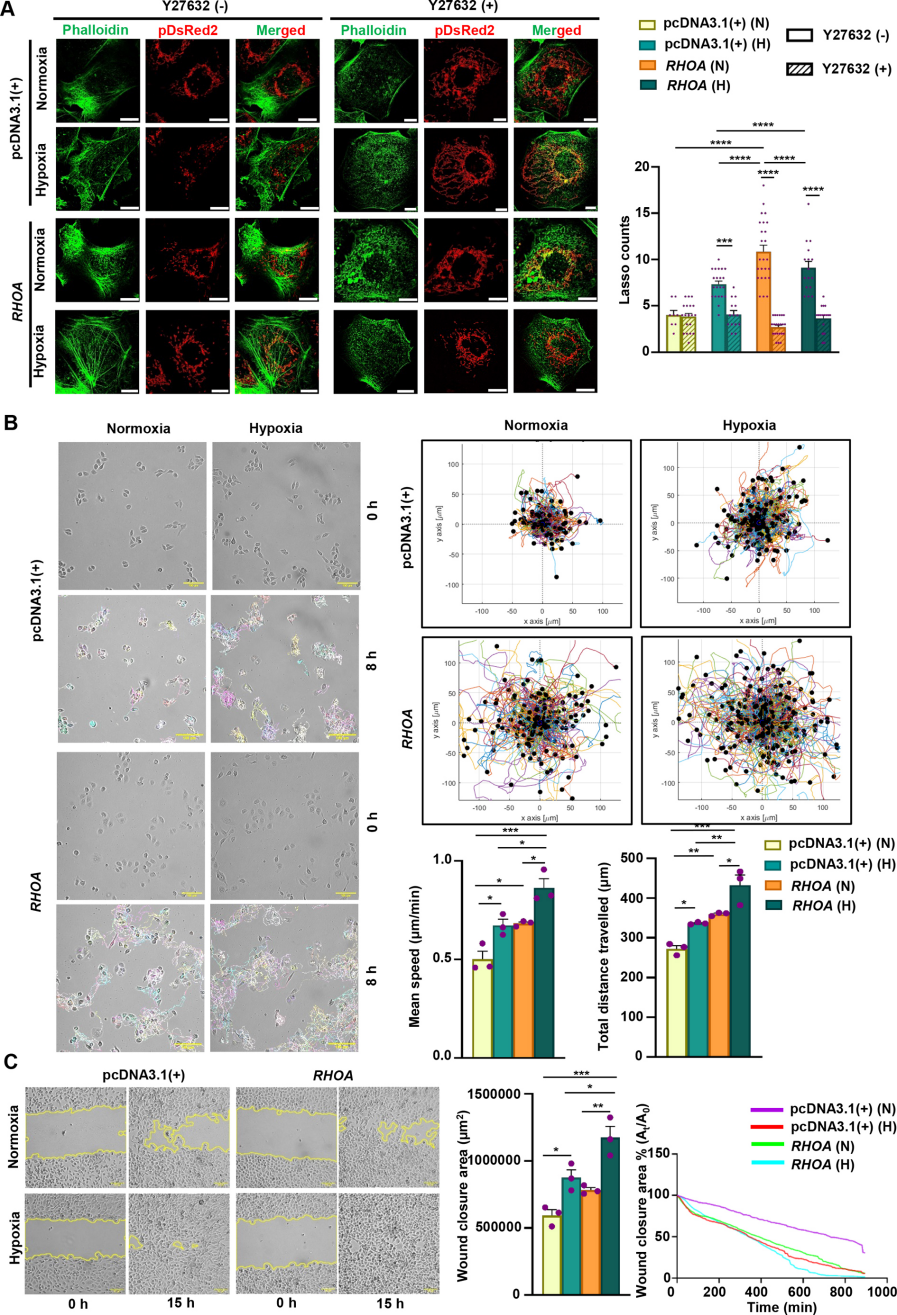
**Mitochondrial morphological changes and GEC motility follow RHOA–ROCK pathway.** (A) Super-resolution microscopic images illustrating the effect of ROCK inhibitor Y27632 on mitochondrial morphology in *RHOA*- and empty vector-stable AGS cells exposed to hypoxia and normoxia. Phalloidin stain was used to represent stress fibres. Objective magnification: 63×. The bar graph (right panel; mean±s.e.m., *n*=3) representing the number of lasso structures (per cell) formed due to the presence or the absence of the ROCK inhibitor. *n*=70 mitochondria/group. Statistical significance was determined by two-way ANOVA followed by Tukey's post hoc analysis. (B) Single-cell tracking analysis showing representative tracks of AGS cells expressing *RHOA* and empty vector for both hypoxic and normoxic environments. Tracking was conducted over an 8 h time period. Single-cell tracks are indicated with a random color. Objective magnification: 10×. Rose plots show the migration tracks of GECs for the above-mentioned conditions. Cell migration from their respective mean positions is indicated by the values on the *x*- and *y*-axes. Bar graphs (mean±s.e.m. from *n*=3) show the mean velocity (μm/min) and of total distance travelled (μm) for those cells that had moved continuously for the 8 h of imaging. Statistical significance was determined by two-way ANOVA followed by Tukey's post hoc analysis. (C) Representative images of scratch assay showing the scratched areas at 0 h and at 15 h in various treatment groups. Objective magnification: 10×. Bar graph (mean±s.e.m. from *n*=3) showing the wound closure area (μm^2^). The continuous plot represents the percentage of wound closure area (A_t_/A_0_) at various time points. Statistical significance was determined by two-way ANOVA followed by Tukey's post hoc analysis. For continuous plot linear regression has been used. In all graphs, individual data points were represented by purple dots. *P<0.05, **P<0.01, ***P<0.001, ****P<0.0001. N, normoxia; H, hypoxia; empty vector, pcDNA3.1(+). Scale bars: 10 μm (A), 100 µm (B,C).

RHO GTPases, ROS levels and cytoskeletal organization are interconnected. Interaction between cell movement and alterations in mitochondrial shape indicates a crucial role for the cytoskeletal system in regulating mitochondrial movement, morphology and perhaps, their functional dynamics (Anesti and Scorrano, 2006). RHOA and its downstream serine/threonine kinase ROCK have been recently known to regulate metastasis in colon cancer cells under the influence of hypoxia (Du et al., 2024), but RHOA–ROCK signaling has not been reported in hypoxic GC cells. The C-terminal of ROCK proteins interacts with active RHOA and phosphorylates downstream effector molecules involved in cytoskeletal rearrangements such as stress fiber, filopodia and lamellipodia formation (Matsuoka and Yashiro, 2014). We became interested to investigate whether the RHOA–ROCK signaling pathway had any role in regulating mitochondrial structural integrity. Therefore, RHOA- and empty vector-stable cells were co-cultured with 40 μM of the ROCK inhibitor Y27632 and exposed to hypoxia and normoxia. The formation of stress fibers was studied using phalloidin to assess the effect of this inhibitor. Treatment with Y27632 led to a reduction in stress fiber formation across all group of cells, indicating effective inhibition of ROCK activity. Additionally, mitochondria adopted a hyperfused morphology, accompanied by a significant decrease in the number of lasso-shaped mitochondria among inhibitor-treated hypoxic empty vector- and normoxic and hypoxic RHOA-stable cells compared to their respective untreated controls (Fig. 6A). In addition to morphological changes, mitochondrial length, circularity and aspect ratio were also influenced due to ROCK inhibition. Y-27632 treatment led to a significant increase in mean branch length and aspect ratio, along with a marked decrease in circularity in hypoxic empty vector-stable cells as well as in both hypoxic and normoxic RHOA-stable cells, compared to their respective untreated controls (Fig. S4A). Next, to quantify the random cell migration under the influence of RHOA and hypoxia, single-cell tracking analysis was performed. This approach allowed for precise measurement of individual cell trajectories within the migrating population, enabling a detailed assessment of how *RHOA* overexpression influenced the coordinated movement of GECs under hypoxic and normoxic conditions. For this, GECs (*n*=75) were tracked for 8 h of continuous hypoxia exposure. The cell movement trajectory was analyzed and represented as a random colorized line (Fig. 6B) and the movement of cells during the entire duration is shown in Movie 5. The positions (*x,y* coordinates) of each tracked cell for a 5 min time interval were used to construct the rose plot. The cumulative plot of the entire 8 h duration demonstrates that RHOA-stable cells had more exploratory movement than empty vector-stable cells. In response to hypoxia, both empty vector- and RHOA-stable cells upregulated the random movement compared to the cells under normoxia with hypoxic RHOA-stable cells having the maximal movement (Fig. 6B). In order to assess the nuanced cellular dynamics, individual cell trajectories were quantified as mean speed and total distance traveled. Interestingly, the mean migration speed and the total distance travelled by RHOA-stable cells were significantly higher than those of the empty vector-stable cells. Under hypoxic conditions, this effect was further amplified, with RHOA-stable cells exhibiting a substantial increase in both mean speed and total distance traveled (Fig. 6B). The change in cell motility was also quantified for 0–3 and 3–8 h time-frames of hypoxic exposure represented as rose plots (Fig. S4B). Up to 3 h, RHOA and empty vector-stable cells did not show noticeable changes in cell motility with or without hypoxia. Surprisingly, cell motility started to increase after 3 h of hypoxia exposure with the maximal motility recorded in hypoxic RHOA-stable cells (Fig. S4B). To elucidate the effect of this RHOA in collective cell migration, a live-wound healing assay was performed for 15 h with image taken at 5 min intervals (Movie 6). The area of the wound decreased over time and the rate at which the wound closed defined the cell migration. The percentages of wound closure area (A_t_/A_0_) were determined. RHOA- and empty vector-stable cells under hypoxia almost completely filled the scratched area within 15 h, whereas normoxic cells showed a healing rate of 50% at 15 h (Fig. 6C). Hypoxic cells also displayed significantly more wound healed area than normoxic ones (Fig. 6C). As compared to overexpressing cells, RHOA-suppressed cells showed reduced motility confirming the role of RHOA in cell movement (Fig. S5A). Additionally, cell motility was studied upon catalase and Y27632 treatment to elucidate the role of ROS and ROCK inhibitor on cell movement. Cell motility decreased upon catalase treatment (Fig. S5B) and ROCK inhibition (Fig. S5C) significantly in RHOA-stable cells but not in empty vector-stable cells. Collectively, these findings establish that RHOA–ROCK transforms mitochondrial shape as well as dynamics and RHOA is crucial in promoting hypoxia-induced GEC motility.

## DISCUSSION

Hypoxia is central in driving tumor progression (Ucaryilmaz Metin and Ozcan, 2022). It deregulates several processes associated with cancer cell motility and stress fiber formation, as well as the energy metabolism of the cell. In the last process, mitochondria are crucially involved and, in response to hypoxia, mitochondria employ strategies to overcome hypoxic stress by modulating their shape and dynamics (Flood et al., 2023). HIF1α-mediated RHOA upregulation has been suggested across multiple types of cancers, including hepatocellular, lung, breast and colorectal cancer (Du et al., 2024; Kataoka et al., 2019; Semenza, 2016; Zhang et al., 2020b). However, contradictory reports exist, where RHOA has been shown as tumor suppressive (Garcia-Mariscal et al., 2018; Kalpana et al., 2019). Although RHOA upregulation occurs in GC and promotes tumor invasiveness (Nam et al., 2019), little is known regarding its function in hypoxic GECs. We were interested to investigate the correlation between RHOA and hypoxia in regulating mitochondrial dynamics and cellular motility in GECs. The present study, reports that elevated RHOA level in response to hypoxia corresponds to a pronounced alteration in mitochondrial dynamics, enhanced ROS generation and increased individual and collective cell motility. Additionally, *RHOA* overexpression induces a shift in mitochondrial morphology, specifically from tubular to lasso. Further investigation unravels the role of ROCK proteins in influencing alteration of mitochondrial morphology. RHOA-induced morphological transformations are hampered in cells treated with either ROCK inhibitor or a ROS scavenger.

A significant increase in RHOA activity at 6 h of hypoxia suggests an early adaptive response to oxidative stress. Interestingly, hypoxic RHOA-stable cells do not further enhance RHOA activity beyond those observed under normoxia, indicating a potential ceiling effect in RHOA activation. A study by Raheja et al. shows that hypoxia decreases RHOA activation in mesenchymal stem cells (Raheja et al., 2011). This suggests that hypoxic influence on RHOA level and activation is highly variable across cell types and culturing conditions. It cannot be generalized that hypoxia consistently leads to elevated RHOA, as a decrease in RHOA level can also occur (Zieseniss, 2014). It should be noted that the time of hypoxic incubation varies immensely between studies. Increasing evidence indicates that RHOA activation during hypoxia is time course regulated (Zieseniss, 2014). Delving into the time-dependent mechanism behind this RHOA-hypoxia interaction would be a fascinating avenue for future research.

Our findings reveal an intricate relationship among RHOA level, hypoxia and mitochondrial structure. Spotting of four distinct mitochondrial morphologies (tubular, lasso, donut and blob) represents a significant advancement in our understanding of mitochondrial plasticity. The predominance of specific shapes under various conditions suggests that mitochondrial morphology serves as a cellular stress response signature. The prevalence of lasso-shaped mitochondria in RHOA-stable cells and hypoxic empty vector-stable cells correlates with elevated RHOA level, confirming the direct influence of RHOA on mitochondrial-reshaping mechanisms. Prior research has largely focused on donut-shaped mitochondria in response to hypoxia, hypoxia-reoxygenation, altered mitochondrial membrane potential alongside the opening of mitochondrial permeability transition pore (m-PTP) and K^+^ ion channels, establishing them as markers of cellular stress (Liu and Hajnóczky, 2011; Long et al., 2015). In our study, the formation of donut-shaped mitochondria predominantly in hypoxic RHOA-stable cells indicates a potential protective response to severe cellular stress, consistent with a previous finding (Liu and Hajnóczky, 2011). This study, for the first time, characterized the lasso shape in normoxic RHOA- and hypoxic empty vector-stable cells, describing a unique Y-shaped tip-to-tip fusion mechanism of lasso formation apart from the previously known tip-to-middle fusion (Preminger and Schuldiner, 2024). The decrease in lasso count in hypoxic RHOA-stable cells might result from the severe stress induced by the combined effects of RHOA- and hypoxia. This suggests that lasso formation serves as a response of mild as well as early stress, which might eventually convert into the donut, allowing cells to optimize energy efficiency in low oxygen condition. Alterations in the ratio of donut to lasso depending on varying degrees of hypoxia and RHOA level could be an interesting addition for follow-up studies.

The correlation between HIF1α and RHOA signaling, and whether HIF1α can regulate the activation of RHOA have not been extensively investigated. This study identifies a significant decrease in RHOA activity upon *HIF1A* suppression, suggesting a potential regulatory role of HIF1α in modulating RHOA signaling. This reduction in activity also alters mitochondrial dynamics from what is observed in case of *RHOA* overexpression. The mitochondria acquire tubular morphology, similar to the normoxic control shRNA-stable cells. However, under hypoxia, *HIF1A*-suppressing cells show a variation in mitochondrial dynamics from normoxic cells, indicating that HIF1α is one of the factors, but might not be the sole factor, responsible for RHOA activation and mitochondrial regulation under hypoxia.

Mitochondrial morphology is related to mitochondrial dynamics (Madan et al., 2021). These dynamic alterations are closely interconnected with ROS generation (Humphries et al., 2023). Enhanced mitochondrial fission is accompanied by elevated ROS. Although these events are extensively studied in breast cancer (Humphries et al., 2023; Lee et al., 2018), there is a lack of information on other types of cancer. In GC, an increase in ROS level is observed in normoxic RHOA-stable cells, which exhibit a higher fission rate. Conversely, reduced ROS levels induce a lower fission rate in hypoxic RHOA-stable cells. These studies align closely with the above findings in breast cancer, reinforcing our observations. This reduction in ROS in RHOA-stable cells under hypoxia might be attributed to the detrimental effects of elevated ROS levels, which can damage proteins and organelles, resulting in significant cellular injury (Nakamura and Takada, 2021). Consequently, *RHOA* overexpression in hypoxic conditions has a protective mechanism that facilitates cancer progression. Hypoxia-induced ROS production not only influences mitochondrial dynamics but also leads to the observed distinct mitochondrial architectures. Our data demonstrate a significant decrease in lasso formation following catalase treatment establishing a mechanistic link between oxidative stress and mitochondrial shape. The RHOA–ROCK pathway is also crucial for maintaining normal mitochondrial morphology (Dong et al., 2023). Treating cells with ROCK-specific inhibitor Y27632 results in hyperfused mitochondria. This suggests that ROCK inhibition might hindered the normal mechanism of mitochondrial shape alterations. Furthermore, hyperfused mitochondria can impair the ability of cells to respond to stressors, contributing to cellular damage and apoptosis (Das and Chakrabarti, 2020).

It is well established that mitochondrial fission increases cell motility and migration in several cancers (Sun et al., 2018; Zhao et al., 2013). Research indicates that loss of mitochondrial fission or increased fusion hinders mitochondrial positioning at the leading edge of a cell thereby limiting cell migration (Madan et al., 2021). Moreover, cell migration is also reduced by decreasing ROS levels (Desai et al., 2013; Wang et al., 2015; Zhao et al., 2013). The exact mechanistic details, however, are mostly unknown, with a few exceptions (Sun et al., 2018; Zhao et al., 2013). In GC, mitochondrial fission has been connected with tumor malignancy, wherein dynamin-related protein 1 (DRP1, also known as dynamin 1-like protein or DNM1L) is the main regulator of mitochondrial dynamics and leads to increased cell motility (Yang et al., 2021). Contradictorily, our study uncovers greater cellular motility in RHOA-stable cells experiencing hypoxia, which exhibit lower fission and ROS. The enhanced exploratory movement and increased migration speed in RHOA-stable cells, particularly under hypoxic conditions, suggest that RHOA activation promotes cellular motility, and hypoxia amplifies this effect through additional signaling pathways. The delayed onset of increased motility, after 3 h of hypoxia, indicates a time-dependent adaptation process. A study by Humphries et al. offers a potential explanation, showing that excessive mitochondrial fission causes actin reorganization, which increases the density of stress fibers, and prevents the development of actin-rich migratory structures and actin-based protrusions at the leading edge of the cell (Humphries et al., 2023). The correlation between mitochondrial shape transformations and cellular behavior (particularly motility) suggests that mitochondrial dynamics might serve as a cellular stress sensor, with RHOA as a key regulator of this response. Consistent with this, RHOA knockdown via siRNA led to a reduction in mitochondrial motility as well as overall cell movement, reinforcing the idea that RHOA-mediated mitochondrial dynamics are essential for promoting cell motility in GC. This study is solely based on an adenocarcinoma cell line. Our findings reflect on cellular invasiveness, which is associated with cancer metastasis. It will be good to explore how metastatic cells behave with respect to mitochondrial alterations and cellular motility driven by RHOA under hypoxia.

In conclusion, this study establishes that RHOA and hypoxia promote mitochondrial lasso formation, involving ROCK proteins, and that the mitochondrial lasso could serve as a marker of early cellular stress. Our research opens the avenue for exploring the time-resolved responses of RHOA to hypoxia in GC, potentially uncovering novel therapeutic targets for tumor development.

## MATERIALS AND METHODS

### Antibodies and reagents

Primary antibodies against α-tubulin (Biobharti, India, #BB-AB0118, rabbit, 1:1000 dilution, western blotting), HIF1α (Abcam, USA, #ab16066, mouse, 1:2000 dilution, western blotting), RHOA (Cell Signaling Technology, USA, #2117S, rabbit, 1:1000 dilution, western blotting) and RHOA (ABclonal, USA, #A0272, rabbit, 1:150 dilution, immunofluorescence microscopy) were used in this study. Secondary antibodies used in this study were anti-rabbit IgG (Cell Signaling Technology, USA, #7074S, 1:2000 dilution, western blotting), anti-mouse IgG (Cell Signaling Technology, USA, #7076S, 1:2000 dilution, western blotting) and Alexa Fluor™ 488-conjugated donkey anti-goat IgG (Invitrogen, USA, #A11055, 1:2000 dilution, immunofluorescence microscopy). Reagents including DAPI (Invitrogen, USA, #D3571, 1:2000 dilution in 1× PBS), 2,7-dichlorodihydrofluorescein diacetate (DCFDA; Sigma-Aldrich, USA, #D6883, 1 µM working concentration), Phalloidin Alexa Fluor™ 488 (Invitrogen, USA, #A12379, 1:400 dilution in 1× PBS), catalase (Sigma-Aldrich, USA, #C9322, 350 units/ml working concentration) and ROCK inhibitor Y-27632 (Sigma-Aldrich, USA, #Y0503, 40 µM working concentration) were used in this study. *RHOA* siRNA and control siRNA (Santa Cruz Biotechnology, USA, # 29471, #37007) were used in some studies. For the RHOA activity assay, cell lysates were prepared using RIPA buffer (HiMedia, India, #TCL131). Mini bio-spin chromatography columns (Bio-rad, USA, #7326207) and glutathione Sepharose 4B GST-tagged protein purification resin (Cytiva, USA, #17075601) were used. GST-tagged RHO-binding domain (RBD) of rhotekin (for pull-down of GTP-bound RHOA), was obtained from Dr Keith Burridge (University of North Carolina, Chapel Hill, NC, USA).

### Cell culture and hypoxia exposure

AGS (ATCC, USA, #CRL-1739) and immortalized non-neoplastic HFE145 (obtained from Duane T. Smoot, Department of Medicine, Meharry Medical Center, Nashville, USA, and Hassan Ashktorab, Department of Medicine, Howard University, Washington, USA) GECs were grown in RPMI 1640 (HiMedia, India) enriched with 10% (v/v) fetal bovine serum (FBS, Gibco, F9665) and maintained in a cell culture flask (T25/T75; Thermo Fisher Scientific) in a humidified incubator (37°C, 5% CO_2_). Both the cell lines are STR-verified. When cell density reached 75–85% confluence, typically after every 2–3 days, cells were passaged using trypsin-EDTA solution (HiMedia, India). For hypoxia, cells were incubated in H35 Hypoxystation (Don Whitley Scientific, UK) and cultured in 3% O_2_, 5% CO_2_ and 92% N_2_ at 37°C with a proper humidified atmosphere.

### Plasmid constructs and stable cell generation

pcDNA3.1(+) (empty vector) (Invitrogen, USA, #V790-20), *RHOA* in pcDNA3.1(+) (Invitrogen, USA, #181860) and pDsRed2-Mito (Takara Bio, Japan, #632421) plasmid constructs were used for stable cell generation. 2×10^4^ AGS cells were initially plated in each well of a 96-well plate with the aim of achieving 70–80% confluence at 24 h prior to transfection. Cells were treated with 10 μl Opti-MEM (Gibco, USA, #31985062), containing 0.2 μg of plasmid, 0.3 μl Lipofectamine 3000 (Invitrogen, USA, #L3000075) and 0.4 μl P3000 (Invitrogen, USA, #L3000075). The medium was changed after 5–6 h. At 24 h post-transfection, transfected cells were selected using G418 solution (Sigma-Aldrich, USA, #A1720, 350 μg/ml working concentration). *HIF1A* shRNA-expressing cells and control shRNA cells were used for *HIF1A* suppression studies, which was prepared as previously described (Bhattacharyya et al., 2010; Rath et al., 2015).

### Transient transfection

RHOA-stable AGS cells were seeded at a density of 10^6^ cells per well in 6-well plates and grown until reaching 70–80% confluence. After 1 day, cells were treated with a transfection mixture (250 μl of Opti-MEM, 2.5 μg plasmid DNA, 7.5 μl Lipofectamine 3000 and 5 μl P3000). The transfected medium was removed after 12 h of incubation and replaced with fresh growth medium. Experiments were performed with the transfected cells at 36–48 h post-transfection. pDsRed2-Mito plasmid construct was used for transient transfection in RHOA-stable and empty vector-stable cells. Similarly, for *RHOA* suppression studies, *RHOA* siRNA and control siRNA were transiently transfected in AGS cells using Lipofectamine 3000 reagent.

### Preparation of cell lysate

10^6^ AGS, empty vector-stable AGS, RHOA-stable AGS and non-neoplastic HFE145 cells were seeded and cultured up to 80% confluence. Cells were then exposed to hypoxia for 6 h and 12 h. After hypoxic treatment, cells were scraped and cell pellets were collected by centrifugation (2000 ***g***, 3 min, 4°C). Each cell pellet was treated with sodium fluoride (NaF) (80 mM; MP Biomedical, France, #194864) and protease inhibitor cocktail (2× PI; HiMedia, India, #ML051-1ML) followed by vortexing until the pellets were completely dissolved in solution. Laemmli buffer (HiMedia, India, # ML021-6ML) and β-mercaptoethanol (5%) (HiMedia, India, #MB041-500ML) mixture was added to the solution and vortexed until pellets were dissolved. Subsequently the samples were heated at 100°C for 8 min followed by storage at −80°C until further use.

### RHOA activity assay

Lysates of AGS cells exposed to 6 h of hypoxia and normoxia were prepared by lysing cells in RIPA buffer and sonicating for 1 min at 50% amplitude with 10 pulses at 5 s intervals using a sonicator (Cole-Parmer, USA). Then lysates were centrifuged for 30 min at 15,000 ***g*** in 4°C. For pull-down experiments, the chromatographic columns were first equilibrated with lysis buffer (50 mM Tris-HCl, 150 mM NaCl, 1% Nonidet P-40, 10 mM MgCl_2_, 1 mM DTT, 2× PI) and then glutathione beads were used at an appropriate amount. After that, cell lysates were incubated with GST-tagged RBD, previously immobilized on glutathione beads for 1 h at 4°C with rotation. Lysates of RHOA-stable and empty vector-stable cells and *HIF1A* shRNA- and control shRNA-stable cells were similarly prepared and incubated with GST-tagged RBD immobilized on glutathione beads. The beads were carefully washed with lysis buffer (each time before and after incubation) and used for immunoblot analysis.

### SDS-PAGE and western blotting

Protein samples were separated using a 12% (v/v) SDS-PAGE resolving gel with a 5% (v/v) stacking gel, run at 130 V for 1 h 45 min in running buffer [25 mM Tris-HCl pH 8.3, 190 mM glycine and 0.1% (w/v) SDS]. Separated proteins were then transferred to 0.45 μm pore-sized nitrocellulose membranes (Merck Millpore, USA, #IPVH00010) at 25 V for 32 min in transfer buffer [25 mM Tris-HCl pH 8.3 190 mM glycine and 20% (v/v) methanol]. Membranes were then washed with 1× TBS (20 mM Tris-HCl, pH 7.6, 137 mM NaCl) for 5 min and blocked with the TBS-T [TBS with 0.1% (v/v) Tween-20] containing 5% non-fat dry milk for 1 h. Following removal of the blocking solution, membranes were exposed to primary antibodies and incubated overnight at 4°C. After overnight incubation, membranes were subjected to 3×10 min washes with TBS-T and incubated with secondary antibodies for 1 h 30 min. Membranes were then washed for 3×5 min with TBS-T and 1×5 min TBS. Protein bands were visualized using ChemiDoc XRS+ system (Bio-Rad, USA) after treating with the ECL Enriched Plus Kit reagents (Abclonal, USA, #RM00021P). See Fig. S6 for uncropped images of blots used in the figures.

### Confocal and immunofluorescence microscopy

5×10^4^–1.5×10^5^ AGS, pcDNA3.1(+)-transfected AGS, RHOA-stable AGS and non-neoplastic HFE145 cells were plated on coverslips in 35 mm cell culture dishes. Following hypoxia exposure, cells were treated with 4% (v/v) paraformaldehyde (PFA; Cat. # P6148) and incubated at 4°C until staining. Then, a series of 1× PBS wash, membrane permeabilization by 0.1% Triton X-100 (v/v) in 1× PBS for 10 min 5% BSA blocking for 1 h and 1× PBST [PBS with 0.1% (v/v) Tween-20] washes for 3 min were done. The primary antibody was diluted in 5% BSA-containing PBST and incubation was done for 12 h at 4°C. Next, an appropriate secondary antibody (1:500 dilution in 5% BSA-containing PBST) was added and incubated in the dark. Nuclear staining was performed with DAPI at 1:2000 dilution in 1× PBS. After 20 min of dark incubation, cells were washed with 1× PBS (3 times, for 5 min each). The coverslips were mounted using mounting medium Fluoromount-G™ (Thermo Fisher Scientific, USA, #00-4958-02). The slides were then allowed to dry in the dark and stored at 4°C until imaging. Images were visualized and captured by either Nikon (Nikon, Tokyo, Japan) or Zeiss (Carl Zeiss, Germany) fluorescence microscopes or Leica DMi8 confocal microscope (Leica, Germany). Captured images were analyzed using NIS-Element Advanced Research software (Nikon) and Fiji (NIH). Fiji macros facilitated the automatic analysis and data extraction.

### Live wound-healing assay and single-cell tracking analysis

Empty vector-stable AGS and RHOA-stable AGS cells were plated densely (1×10^5^–2×10^5^ cells per plate) or sparsely (3×10^4^–5×10^4^ cells per dish) in 35 mm cell culture dishes for the live wound-healing assay (collective migration) and single-cell tracking analysis (random migration), respectively. For the live wound-healing experiment, cells were grown until 80–90% confluency. Cells were exposed to hypoxia. Post-hypoxic exposure, wound marks were created, and cells washed twice to remove the cell debris or floating cells. Wound closure was tracked, and images were taken every 5 min until complete closure was achieved. For random migration, cells were cultured until settled. Cells were tracked with exposure to hypoxia (3% O_2_, 5% CO_2_ and 92% N_2_ at 37°C) and normoxia (16% O_2_, 5% CO_2_ and 77% N2 at 37°C). Images were captured every 5 min for 8 h. For both migration assays, cells were tracked with CytoSMART Lux3™ BR bright field live-cell imager (Axion Biosystem). For image analysis, initially cells were segmented by using Cellpose 2.0 (Pachitariu and Stringer, 2022) under BIOP plugin in Fiji. The image segmentation process was done automatically with the help of the Fiji macro. Then, the segmented images were tracked with TrackMate (Ershov et al., 2022). The wound closure area was calculated and labeled automatically with the wound_healing_size_tool (Suarez-Arnedo et al., 2020).

### Super-resolution microscopy and mitochondrial dynamics

5×10^4^–1×10^5^ pDsRed2-Mito-stable AGS cells, pDsRed2-Mito-transfected RHOA-stable AGS cells were seeded in 35 mm or confocal 35 mm dish (SPL Life Sciences, South Korea, #200350) 24 h before fixing and live imaging, respectively. Cells were exposed to hypoxia for an appropriate time period. Following hypoxic exposure, live-cell super-resolution imaging was done by using a Zeiss LSM 880 Airyscan super-resolution system (Carl Zeiss, Oberkochen, Germany) equipped with a proper incubation chamber (5% CO_2_, 37°C). Time-lapse images were taken continuously with an interval of 5 s for 3 min. Raw data images were then processed and converted into SIM images with the help of ZEN (black edition) software (Carl Zeiss Microscopy, elyra7). For mitochondrial dynamic analysis, an ROI was first selected for at least 20–25 mitochondria. Nellie (Lefebvre et al., 2025), an automated organelle segmentation and tracking plugin in Napari, was used to acquire the mitochondrial dynamics data. For fixed imaging, coverslips were prepared as explained in the ‘confocal and immunofluorescence microscopy’ section above. *Z*-stack images were acquired and reconstituted into volumetric 3D images in Zeiss arivis Vision4D or ZEN blue 3.0 software (Carl Zeiss Microscopy, elyra7).

### Cellular ROS detection and catalase treatment

5×10^4^–1×10^5^ empty vector-stable AGS and RHOA-stable AGS cells were seeded on coverslips in 35 mm cell culture dishes. Cells were treated with or without 350 units/ml catalase 1 h prior to hypoxia exposure (Dixit et al., 2022). After 5 h of hypoxia exposure, 1 µM DCFDA was added. Incubation was undertaken for 1 h at 37°C, maintaining hypoxia, in complete dark conditions to avoid photobleaching. Coverslips were fixed with 4% (v/v) PFA (Sigma-Aldrich) in PBS followed by incubation in dark condition at 27°C. DAPI (1:2000 dilution in 1× PBS) was used for nuclear staining followed by 20 min of dark incubation. Coverslips were washed with 1× PBS (3 times, for 5 min). The mounting of coverslips was done with Fluoromount-G™ mounting medium. The slides were then allowed to dry in the dark and stored at 4°C till imaging. Images were captured from three independent fields of each group with a Nikon Eclipse TiU/Eeclipse Ni-E fluorescence microscope. ROS level was detected and quantified automatically by using Fiji macro.

### Bioinformatics analysis

The TIMER2.0 database (http://timer.cistrome.org/) and the UALCAN database (https://ualcan.path.uab.edu/) were used to present RNA-seq and all-inclusive gene expression datasets that can be easily evaluated between normal and cancer tissues based on various samples. The KM plotter (https://kmplot.com/analysis/), a free public software, was used to assess the prognosis regarding varied protein-related conditions and also provided a comprehensive survival analysis of any diseased or diseased free individuals. Samples were categorized into high-expression and low-expression groups using the median value as the threshold, with a 50% high and low cut-off value. GEPIA2 (http://gepia2.cancer-pku.cn/) was used to determine the correlation analysis. The gene expression patterns across various cell types in STAD were analyzed using data from the HPA (https://www.proteinatlas.org/), which is a comprehensive database of protein expression in human tissues and cells.

### Statistical analysis

Statistical analyses were performed using GraphPad Prism software (Version 9; GraphPad Software Inc, CA, USA). Unpaired two-tailed *t*-tests, linear regression, and one-way and two-way ANOVA followed by Tukey's post hoc analysis were applied to determine the statistical significance. All the bar graphs, continuous graphs and cell trajectory plots were generated using GraphPad and MATLAB. Statistical significance (*P* value) is denoted by star annotations.

## Supplementary Material

10.1242/joces.263690_sup1 Supplementary information
