# Supplementary material for: RHOA-dependent regulation of mitochondrial remodeling and cell motility in hypoxia-exposed gastric epithelial cells
Source: J Cell Sci. 2025 Jul 30;138(14):jcs263690. doi: 10.1242/jcs.263690 (PMC12377718; doi:10.1242/jcs.263690)
Supplement: Supplementary information [file joces-138-263690-s1.pdf]

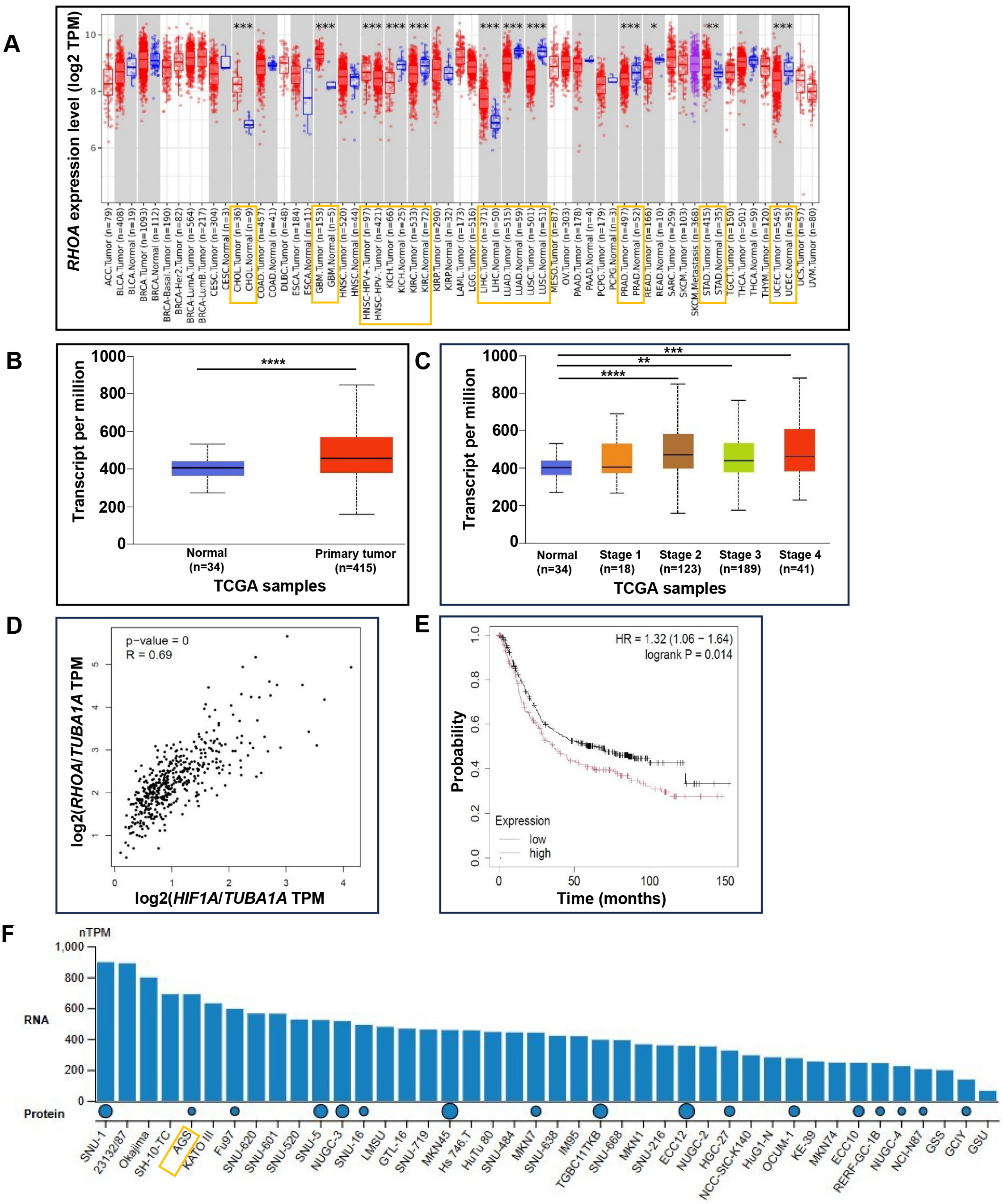

Fig. S1. *RHOA* expression is enhanced in GC and is related to *HIF1A*.

(A) TIMER2.0 analysis showing differential *RHOA* expression in various tumor (red) and normal (blue) samples. Significant expression have been highlighted with yellow boxes. (B-C) Graphs showing expression analysis of *RHOA* transcript using UALCAN web-portal in normal and primary tumors and in subgroups based on stages of STAD. STAD=Stomach adenocarcinoma. **P<0.01, ***P<0.001, ****P<0.0001. (D) Correlation analysis of *RHOA* and *HIF1A* normalized with *TUBA1A* in STAD and normal TCGA samples using GEPIA2. The correlation was given in the form of p-value. P=Pearson coefficient. (E) Kaplan-Meier plot depicting the probability of survival over time due to high (red line) and low expression (black line) of *RHOA*. (F) Bar graph illustrating the RNA and protein level of RHOA in 42 GC cell lines with AGS marked with a yellow box. nTPM=690.5 (RNA) and nRPX=0.0741(protein). TPM=Transcripts per million.

Journal of Cell Science • Supplementary information

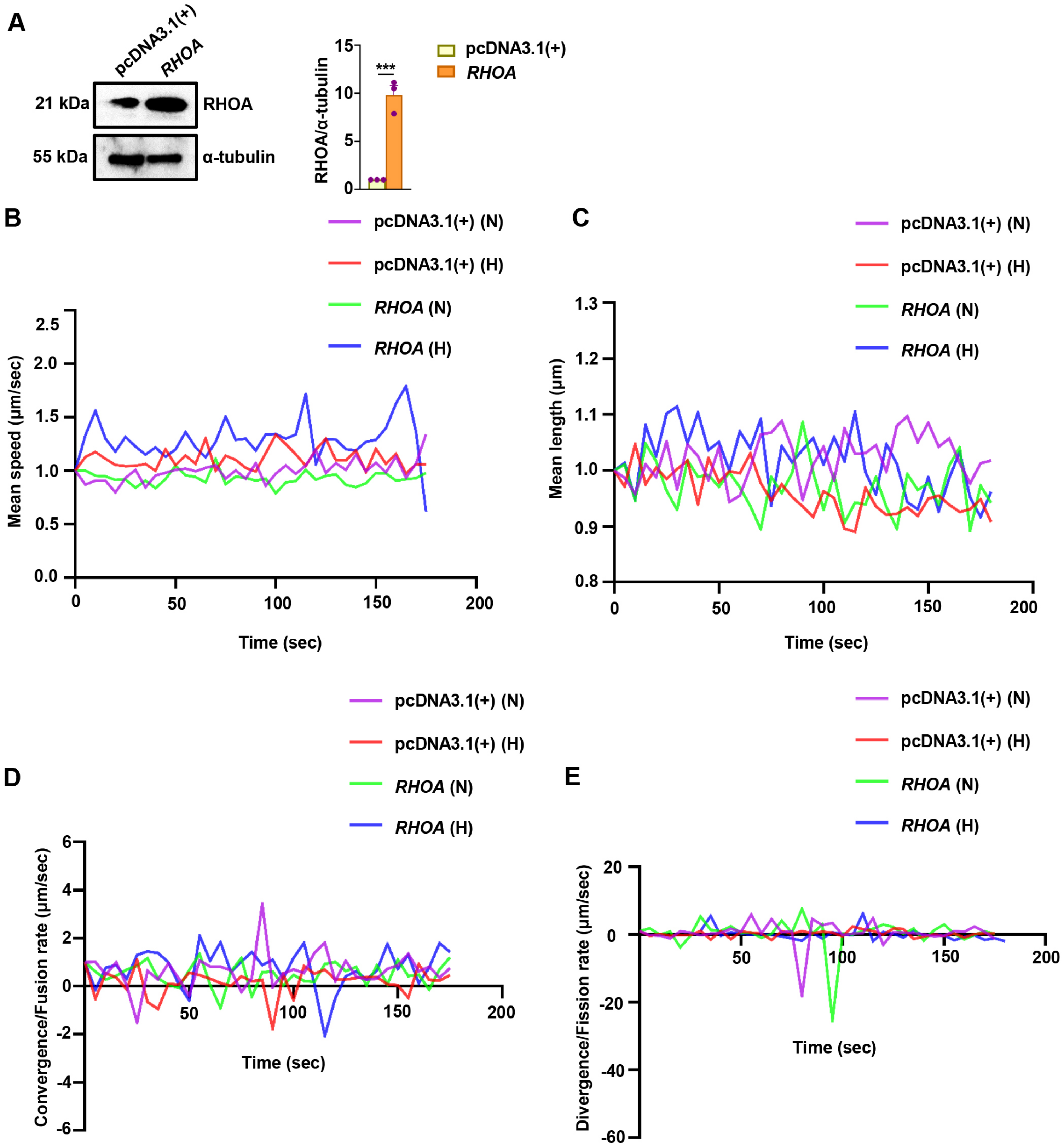

Fig. S2. Mitochondrial dynamics change in response to RHOA and hypoxia. (A) Western blot comparing RHOA protein in AGS cells transfected with *RHOA*-overexpression plasmid and empty vector-stable cells. α-tubulin was taken as loading control. Quantification of the RHOA band relative to α-tubulin has been shown in the bar graph (mean ± sem, n=3). Statistical significance was determined by two-way ANOVA followed by Tukey's post hoc analysis. ***P < 0.001. Individual data points were represented by purple dots on bars. A representative plot (mean ± sem, n=3) depicting the continuous fluctuations of (B) mean speed, (C) mitochondrial length, (D) convergence/ fusion rate and (E) divergence/ fission rate within 180 sec in RHOA and empty vector-stable cells exposed to hypoxia and normoxia. Statistical analysis was done by using linear regression (curve fit). N= Normoxia; H= Hypoxia.

Journal of Cell Science • Supplementary information

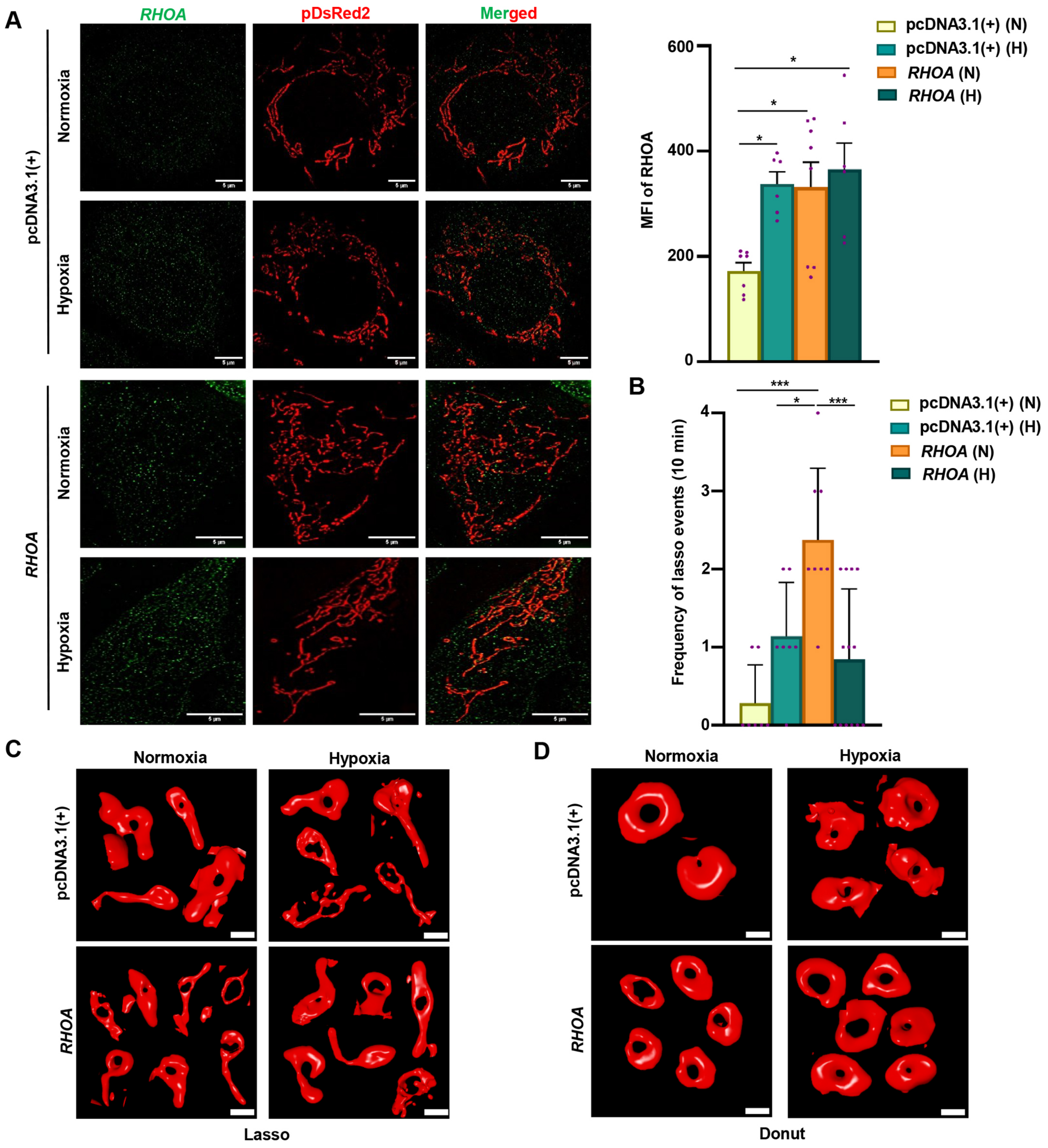

Fig. S3. Lasso shape increases with increased RHOA level. (A) Super-resolution micrographs illustrating mitochondrial morphology changes in *RHOA* and empty vector-stable AGS cells with or without hypoxia. RHOA was stained green with a RHOA-specific antibody. Scale bar: 5 µm, Objective magnification: 63×. The bar graph (mean ± sem, n=3) showed the MFI of the four groups. n>100 mitochondria/ group. Statistical significance was determined by two-way ANOVA followed by Tukey's post hoc analysis. MFI= Mean fluorescence intensity. (B) Representative graph (mean ± sem, n=3) showing the frequency of lasso events within a 10 min time-frame in the same cell groups. Statistical significance was determined by two-way ANOVA followed by Tukey's post hoc analysis. Individual data points were represented by purple dots on bars. (C-D) Figures depicting 3D-models of various lasso and donut shapes found in empty vector or RHOA-stable cells subjected to hypoxia and normoxia. Scale bar: 0.5µm. *P < 0.05, ***P < 0.001. N= Normoxia, H= hypoxia.

Journal of Cell Science • Supplementary information

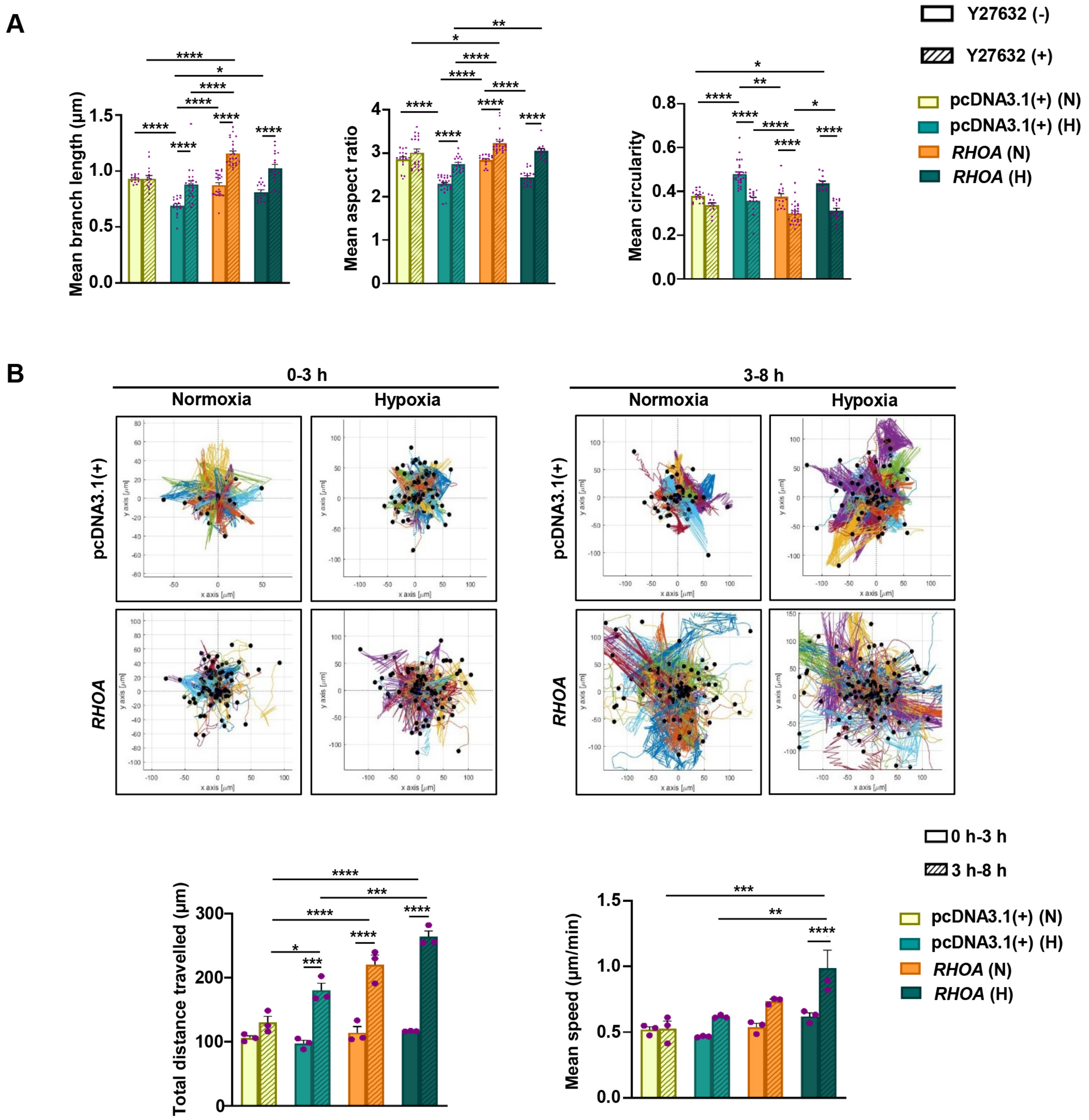

Fig. S4. Mitochondrial morphology, cell motility and wound closure properties are regulated by RHOA and hypoxia. (A) Graphical representations (mean ± sem, n=3) showing the effect of ROCK inhibitor Y27632 on mitochondrial mean branch length, circularity and aspect ratio in RHOA and empty vector-stable AGS cells exposed to hypoxia and normoxia. n=70 mitochondria/ group.(B) Rose plot showing the migration tracks of AGS cells transfected with RHOA and pcDNA3.1+ under both hypoxic and normoxic conditions for 0-3 h and 3-8 h respectively. The values on X- and Y-axis denote the extent of migration by cells from their respective mean positions. Bar graph (mean ± sem from n=3) showing the mean speed (µm/min) and of total distance travelled (µm) by AGS cells transfected with *RHOA* and pcDNA3.1+ exposed to hypoxic and normoxic conditions for 0-3 h and 3-8 h. (C) Bar graph (mean ± sem from n=3) showing the wound closure area (µm2) after 15 h in RHOA and pcDNA3.1+ expressing AGS cells under normoxia and hypoxia. Statistical significance was determined by two-way ANOVA followed by Tukey's post hoc analysis. *P < 0.05, **P < 0.01, ***P < 0.001, ****P < 0.0001. Data points have been indicated as purple dots in the bar graphs. N= Normoxia, H= Hypoxia.

Journal of Cell Science • Supplementary information

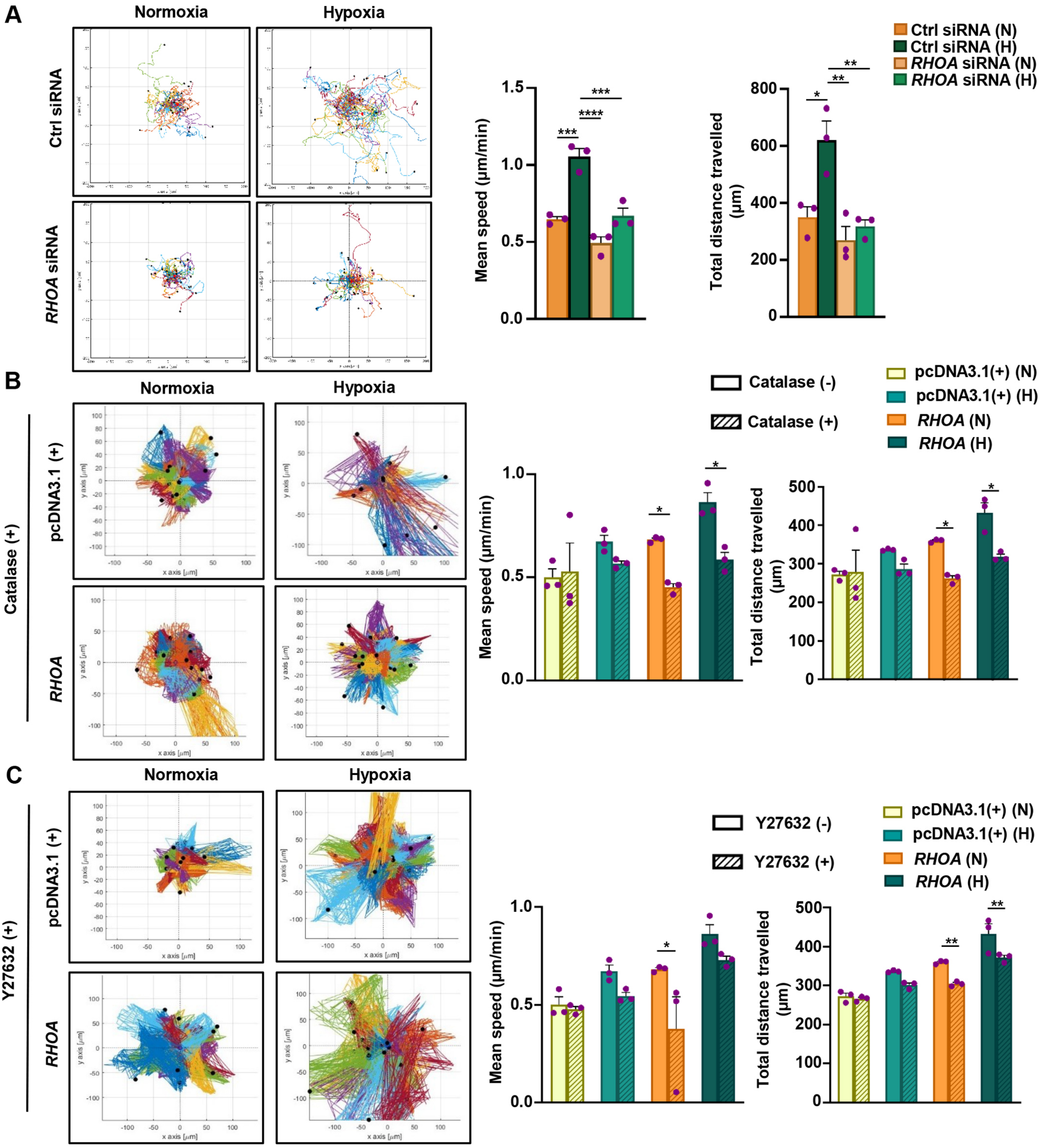

Fig. S5. *RHOA* suppression, ROS scavenging, and ROCK inhibition reduces single-GEC motility. Representative rose plots depicting the exploratory behavior of GEC cell motility following suppressing RHOA (A), catalase treatment (B) and inhibiting ROCK activity (C). Bar graphs (mean ± SEM) summarize migratory parameters, including mean speed and total distance travelled. Statistical significance was determined by two-way ANOVA followed by Tukey's post hoc analysis. *P < 0.05, **P < 0.01, ***P < 0.001, ****P < 0.0001. Individual data points were represented by purple dots. N= Normoxia, H= Hypoxia.

Journal of Cell Science • Supplementary information

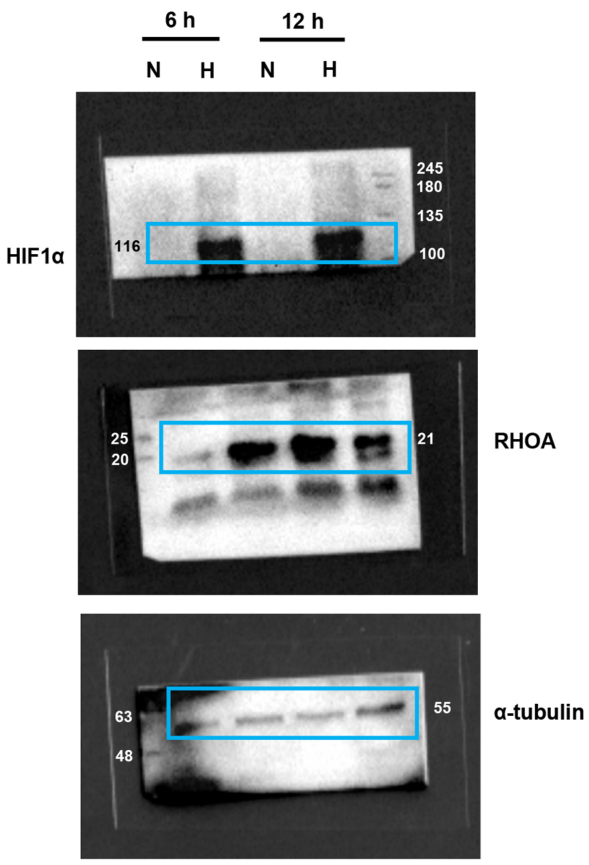

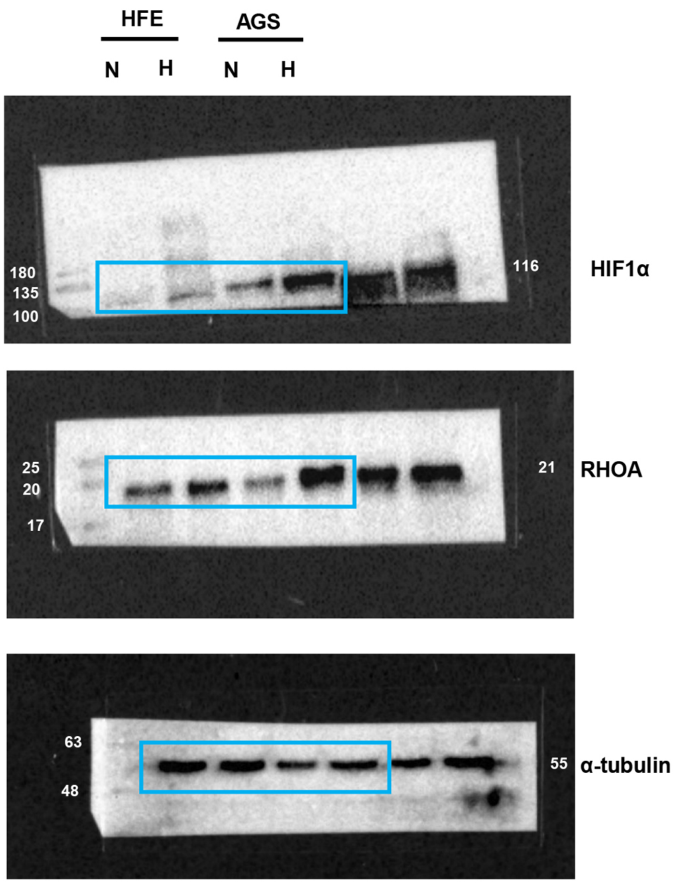

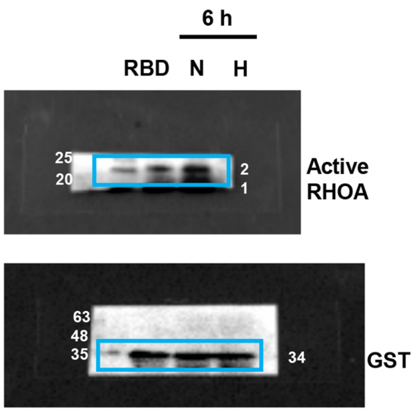

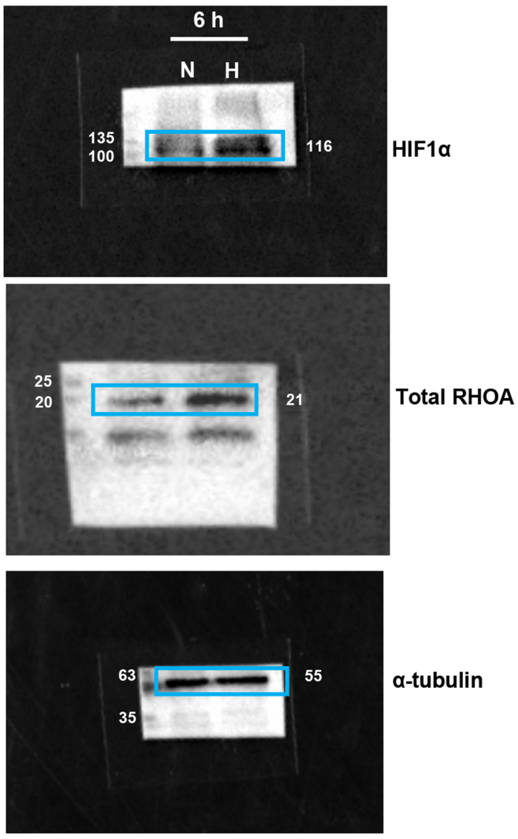

Journal of Cell Science • Supplementary information

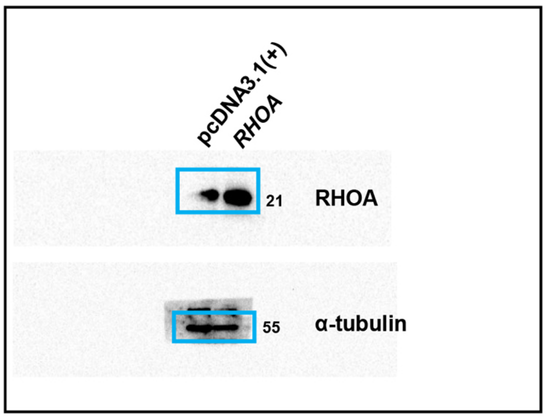
pcDNA3.1(+)
RHOA
21
RHOA
55
α-tubulin

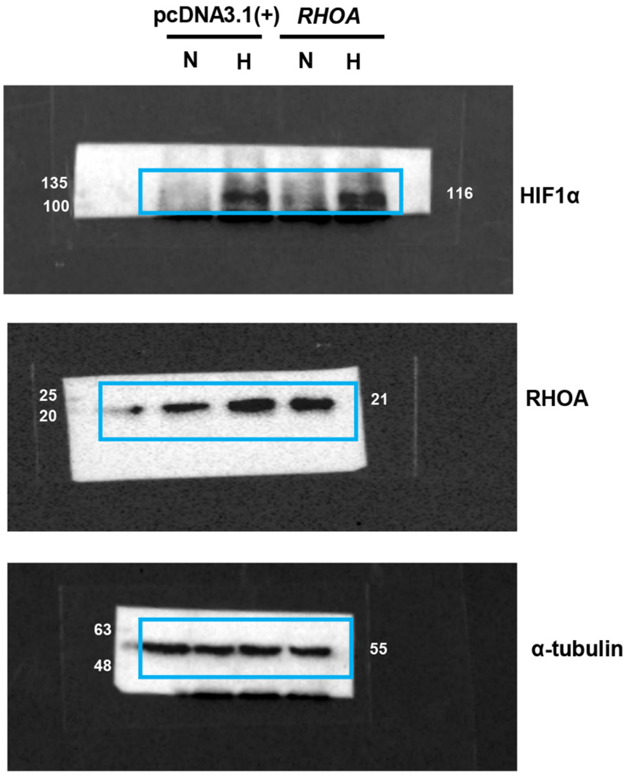
pcDNA3.1(+)
RHOA
N H N H
135
100
116
HIF1α
25
20
21
RHOA
63
48
55
α-tubulin

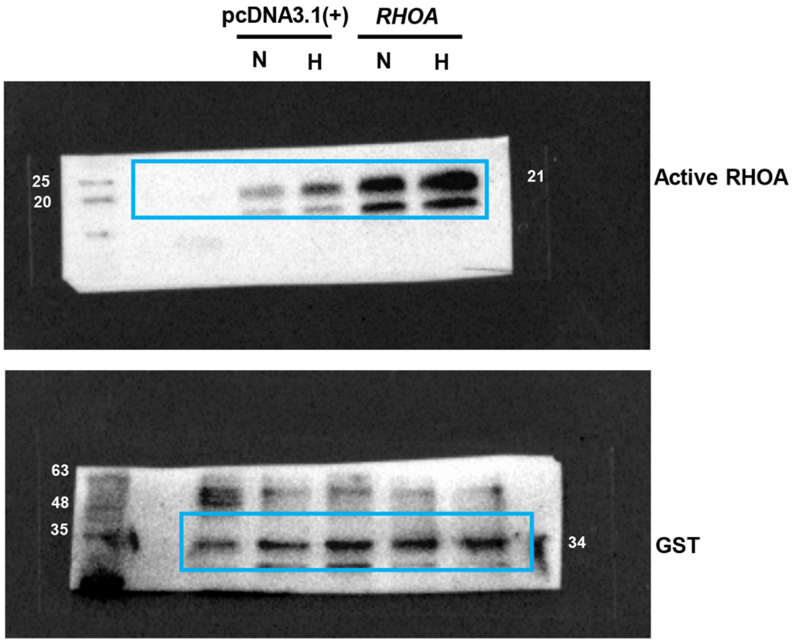
pcDNA3.1(+)
RHOA
N H N H
25
20
21
Active RHOA
63
48
35
34
GST

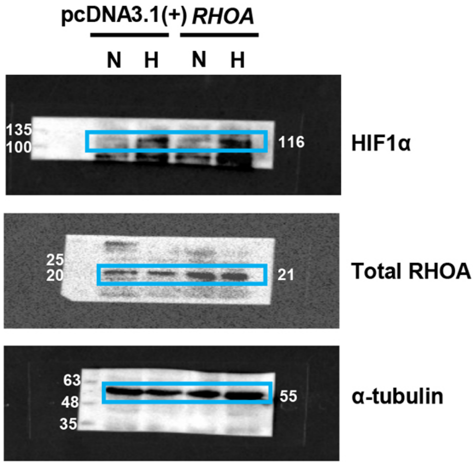
pcDNA3.1(+)
RHOA
N H N H
135
100
116
HIF1α
25
20
21
Total RHOA
63
48
35
55
α-tubulin

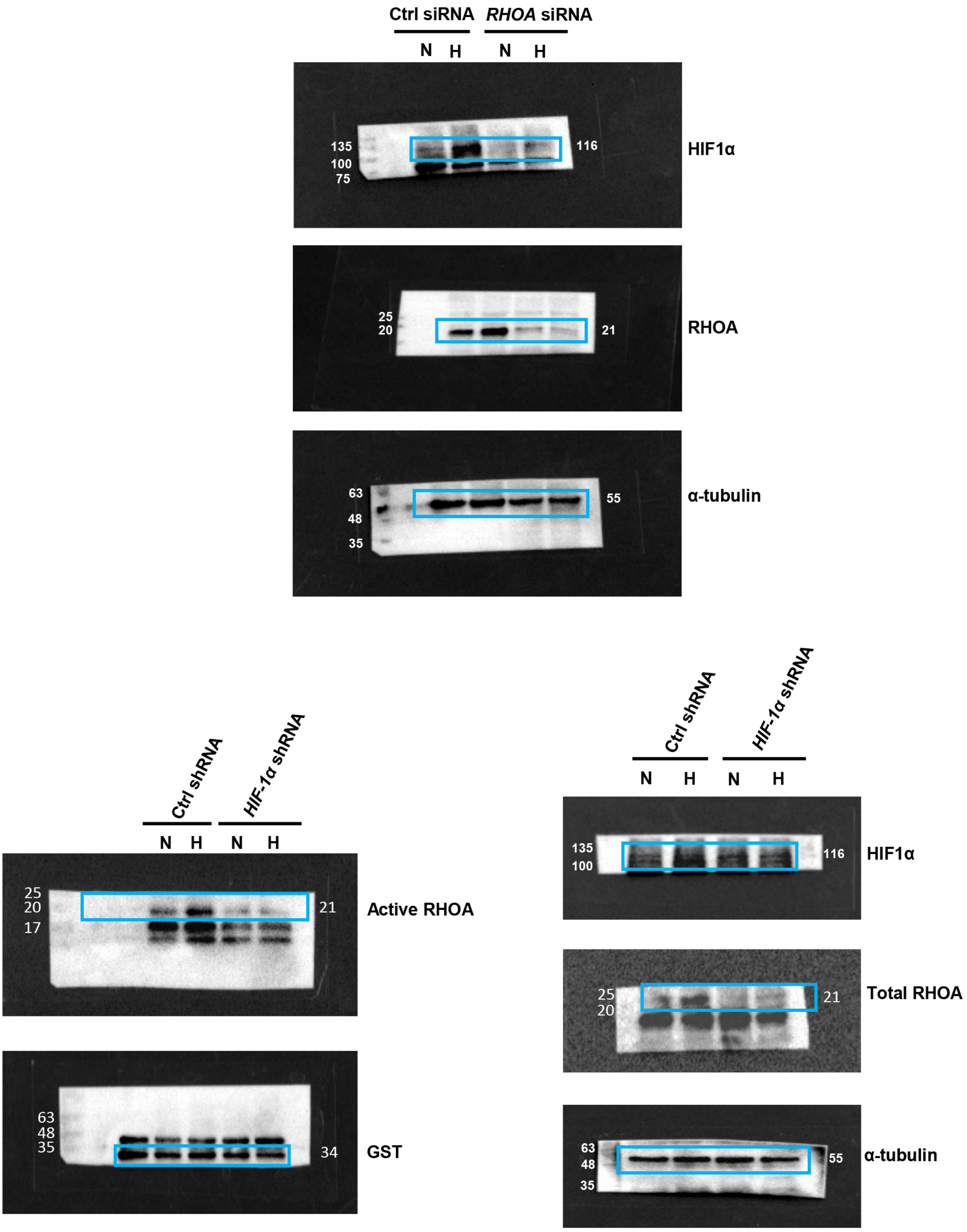

Fig. S6. Blot Transparency. Uncropped images of the blots corresponding to all main and supplementary figures are shown. The regions used in the figures are highlighted with blue boxes.

Journal of Cell Science • Supplementary information

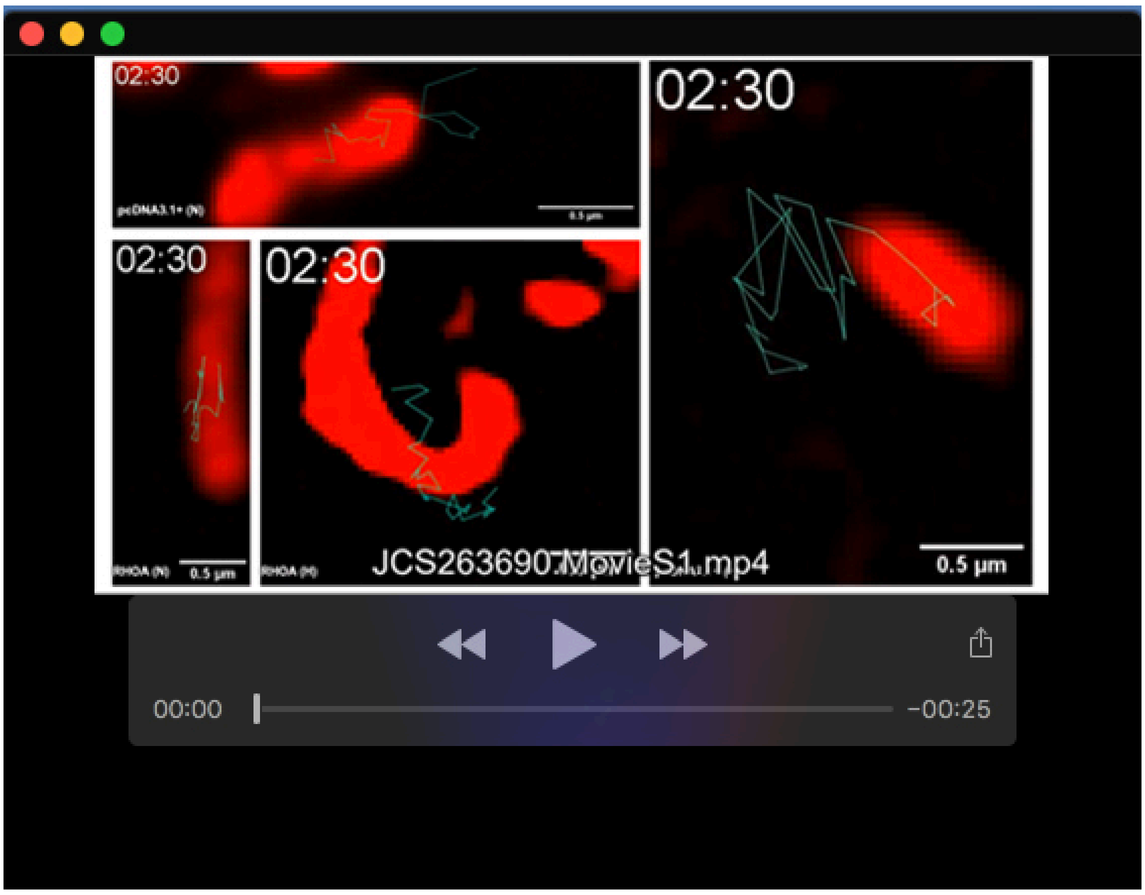

Movie 1. Time-lapse movie of mitochondrial speed in *RHOA* and empty vector-transfected AGS cells exposed to hypoxia and normoxia, related to Fig. 2C. The time-lapse was obtained for 180 sec with 5 sec intervals using a super-resolution microscope. Scale bar: 0.5 µm. Display rate = 10 frames per sec (FPS).

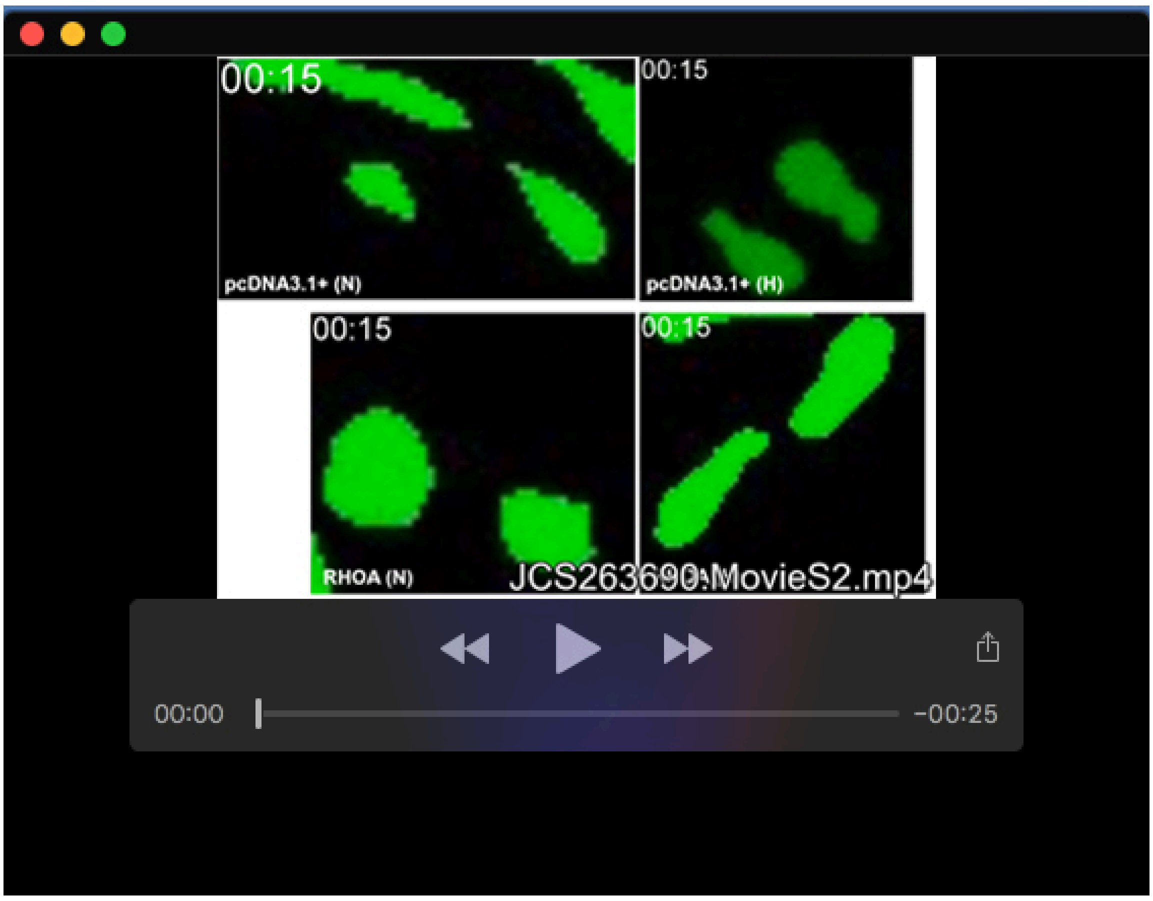

Movie 2. Time-lapse movie of mitochondrial fission in hypoxia-exposed *RHOA* and empty vector-expressing AGS cells related to Fig. 2D. The time-lapse was shown for 25 sec with 5 sec intervals using a super-resolution microscope. Display rate = 1.5 FPS.

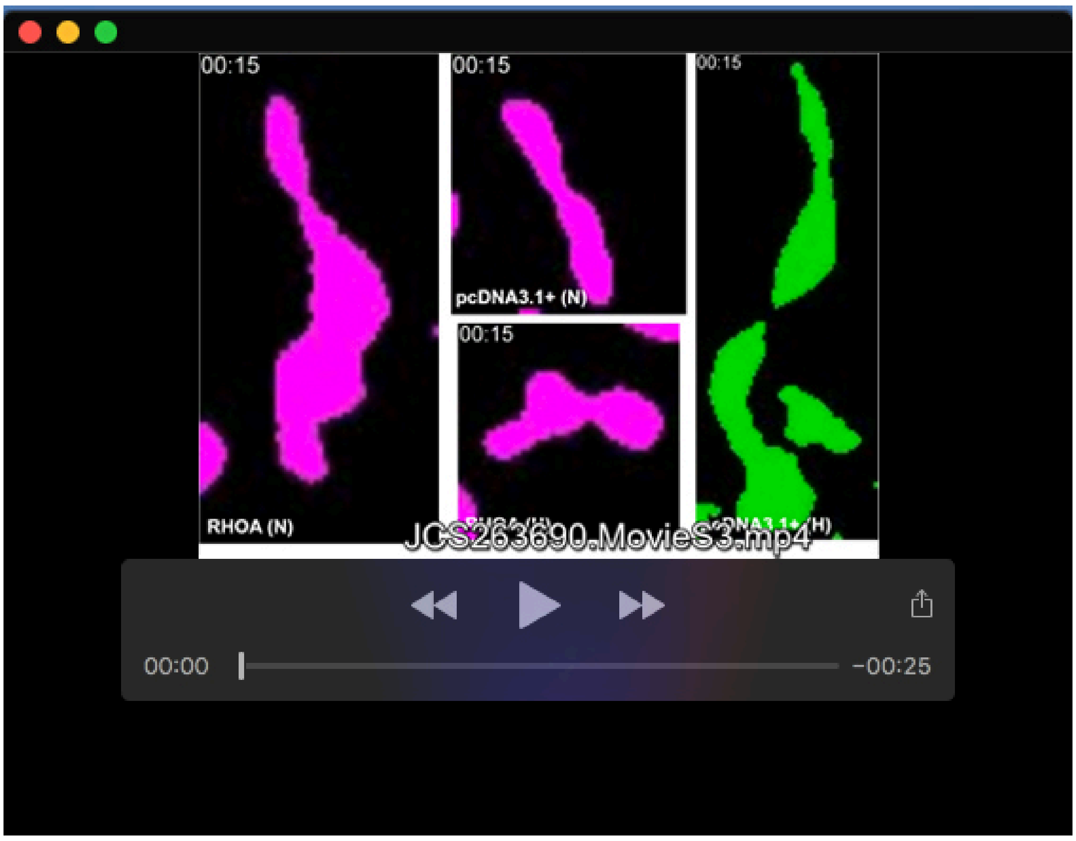

Movie 3. Time-lapse movie of mitochondrial fusion in *RHOA* and empty vector-expressing AGS cells subjected to hypoxia and normoxia related to Fig. 2D. The time-lapse was shown for 25 sec with 5 sec intervals using a super-resolution microscope. Display rate = 1.5 FPS.

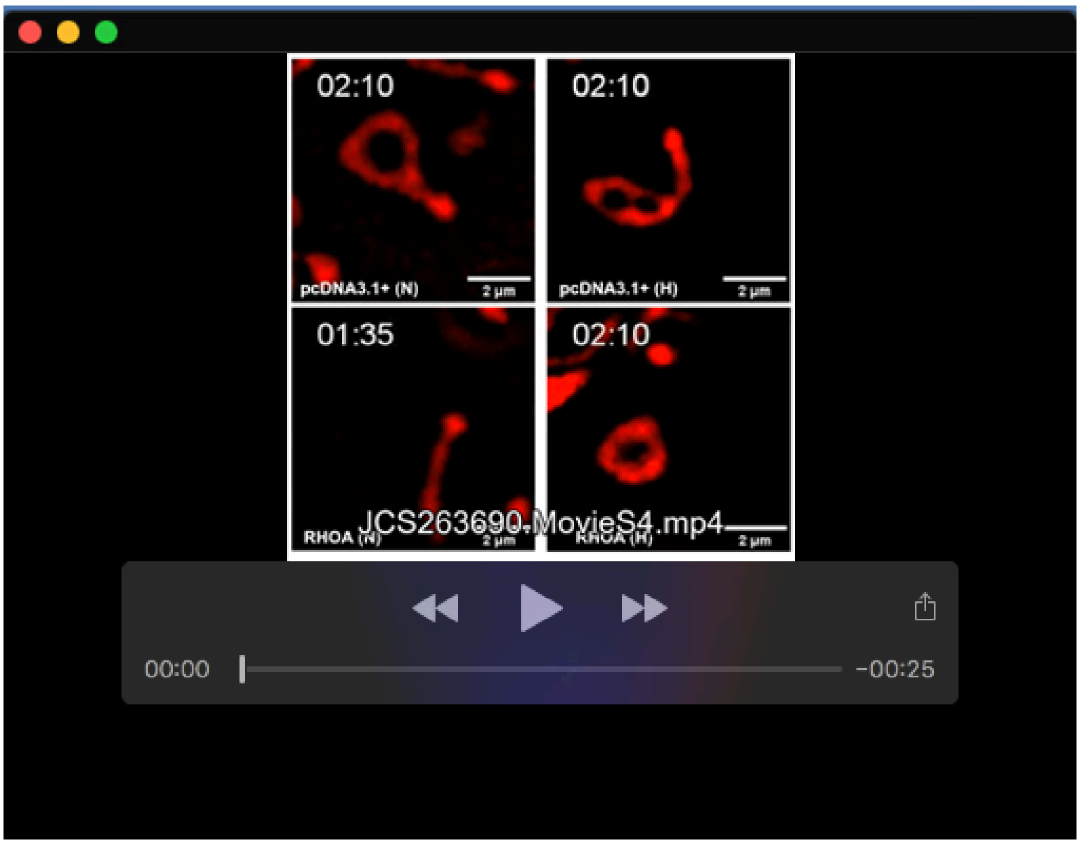

Movie 4. Time-lapse movie of mitochondrial lasso formation in *RHOA* and empty vector-expressing AGS cells exposed to hypoxia and normoxia, related to Fig. 3B. The time-lapse was obtained for 10 min with 5 sec intervals using a super-resolution microscope. Display rate = 5 FPS.

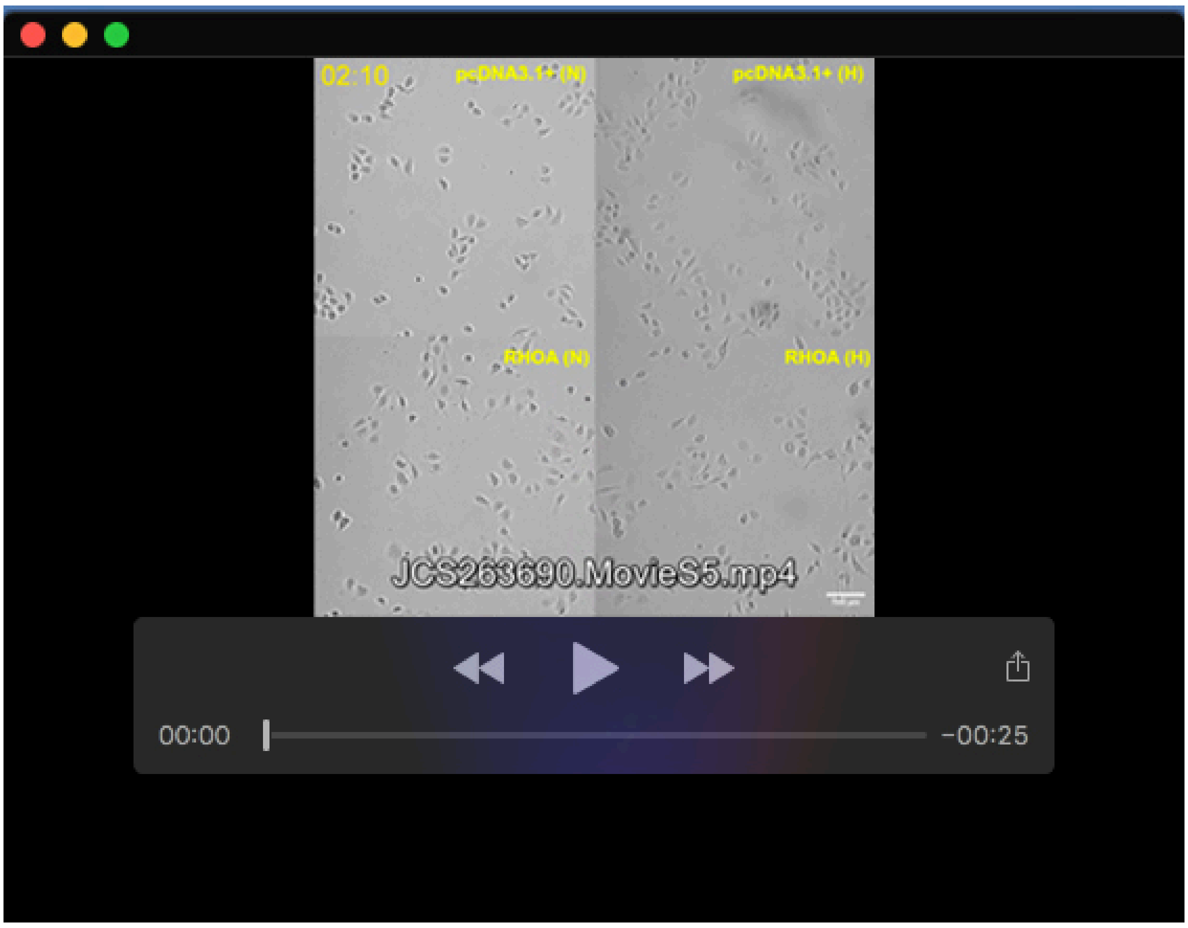

Movie 5. Time-lapse movie of single cell movement in *RHOA* and empty vector-expressing AGS cells under hypoxia and normoxia related to Fig. 6B. The time-lapse was generated for 8 h with 5 min intervals using cytosmart. Display rate = 20 FPS.

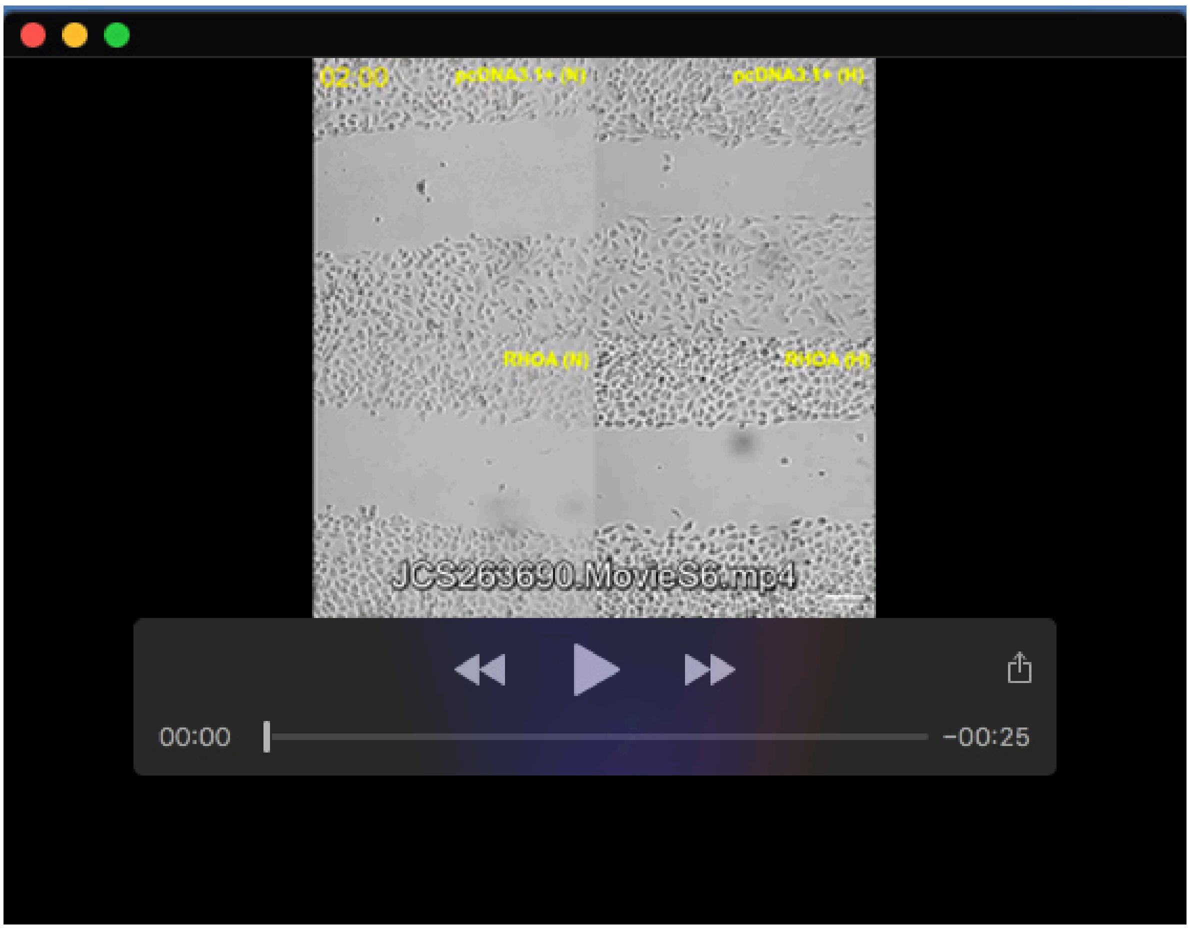

Movie 6. Time-lapse movie capturing the migration of *RHOA* and empty vector-expressing AGS cells under hypoxia and normoxia in wound healing assay related to Fig. 6C. The time-lapse was generated for 15 h with 5 min intervals using cytosmart. Display rate = 20 FPS.

Journal of Cell Science • Supplementary information
